# The novel link between planar möbius aromatic and third order nonlinear optical properties of metal–bridged polycyclic complexes

**DOI:** 10.1038/s41598-017-10739-7

**Published:** 2017-08-31

**Authors:** Li Wang, Jinting Ye, Hongqiang Wang, Haiming Xie, Yongqing Qiu

**Affiliations:** 1 Institute of Functional Material Chemistry, Faculty of Chemistry, Northeast Normal University, Changchun, 130024 China; 2 National & Local United Engineering Laboratory for Power Battery, Faculty of Chemistry, Northeast Normal University, Changchun, 130024 China

## Abstract

Metal–bridged polcyclic aromatic complexes, exhibiting unusual optical effects such as near-infrared photoluminescence with particularly large Stokes shifts, long lifetimes and aggregation enhancement, have been established as unique “carbonloong chemistry”. Herein, the electronic structures, aromaticities, absorption spectra and third order nonlinear optical (NLO) responses of metal–bridged polcyclic aromatic complexes (M = Fe, Re, Os and Ir) are investigated using the density functional theory computations. It is found that the bridge–head metal can stabilize and influence rings, thus creating π–, σ– and metalla–aromaticity in an extended, π–conjugated framework. Interestingly, metal radius greatly influence the bond, aromaticity, liner and third order NLO properties, which reveals useful information to develop new applications of metal regulatory mechanism in NLO materials field. Significantly, the novel relationship between the aromaticity and third order NLO response has firstly been proposed, that the metal-bridged polycyclic complex with larger aromaticity will exhibit larger third order nonlinear optical response. It is our expectation that the novel link between aromaticity and NLO response could provide valuable information for scientists to develop the potential NLO materials on the basis of metal–bridged polycyclic complexes.

## Introduction

**Figure 1:**
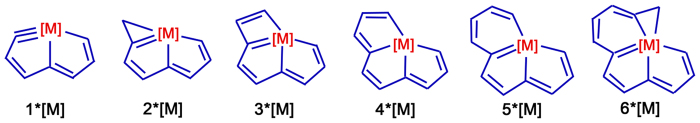
The structures of metalla-aromatic molecules.

Aromaticity is still one of the central and compelling topics that has long interested both experimentalists and theoreticians because of its fascinating and constantly growing diversity^1–4^. The Hückel aromaticity rule^5, 6^ applies to cyclic circuits of 4n + 2 mobile electrons^4^, yet Craig–Möbius topologies favour 4n π–electron conjugation counts^7–9^. The seminal proof-of-concept work on Craig-Möbius aromaticity in planar metallacycles was reported by Mauksch and Tsogoeva^9^. It first presented the building principles for Craig-Möbius aromatics and with transition metals from group 8 (Fe, Ru, Os). Mauksch and Tsogoeva first solved the problem of distinguishing Möbius from Hückel aromaticity by establishing the 4n electron count by inspection of the orbitals^9^. The realization of small cyclic alkynes challenges synthetic chemists because the angle strain associated with the highly distorted triple bonds must be overcome^10–14^. Fortunately, the introduction of a metal fragment is an efficient strategy to stabilize cyclic alkynes by reducing ring strain^15–25^. Metalla–aromatic molecules, first proposed by Thorn and Hoffmann, are analogues of conventional aromatic molecules, in which one carbon segment is formally replaced by a transition–metal fragment^7^. Bicyclic metallapentalynes (1*[Os], Fig. 1) was achieved by means of the incorporation of the osmium centre not only reduces the ring strain of the parent pentalyne, but also converts its Hückel antiaromaticity into Möbius aromaticity^26^. In addition to the π–aromaticity, σ–aromaticity was also observed in an unsaturated metallacyclopropene unit of cyclopropametallapentalenes (2*[Os])^27^. Metal–bridged tricyclic aromatic complex (3*[Os]) was reported through the stabilization of two typically antiaromatic frameworks (cyclobutadiene and pentalene) by introducing a metal^28^. Soon after, another metal–bridged tricyclic aromatic system was also synthesized, in which the metal center is shared by three aromatic five–membered rings^29^. In this work, the oxygen and nitrogen atom in the literature was replaced with carbon atoms to obtain the complex 4*[Os]. Then, 6*[Os] was surrounded with the completion of a metal–bridged polycyclic aromatic system, in which all of the five coordinated carbons lie in the equatorial plane. It is the largest planar Möbius aromatic system synthesized to date^30–33^. Hereto, a whole new aromatic system as well as the unique “carbonloong chemistry” have been established. Although the aromaticities of these metal–bridged polcyclic aromatic complexes have been studied, other properties such as the bonding properties, optical properties, etc, have not attracted much attention.

The third order nonlinear optical (NLO) properties of organic π–conjugated compounds have been extensively investigated because of their flexible molecular design and potentially low processing cost^34^. The link with NLO properties has been deduced for several parameters including the length of the π–conjugated linkers^35^, the charge^36^, the shape and the dimensionality of the π–electron network^37^ and the donor/acceptor substituents^38^. However, the relationship between the aromaticity and NLO response was rarely studied^39^. In this work, continuing our research on NLO response, we would carry out the density functional theory (DFT) computations on complexes 1–6*[M] (M = Fe, Re, Os and Ir). Systematic studies of bond properties between M and C, aromaticity, spectroscopic and NLO properties would be performed. The particular aim of the present paper is to propose the novel relationship between the aromaticity and NLO response.

## Method

The structural optimization calculations for the complexes are carried out using B3LYP functional with def2-TZVPP basis set^40^. UV/Vis absorption spectra were calculated with two kinds of polarizable continuum model (PCM) solvents (dichloromethane and methanol) at TD-B3LYP/def2-TZVPP^41^. All of the calculations were carried out by using the Gaussian 09 W program package^42^. Localized-orbital locator (LOL), a bond descriptor based on the kinetic-energy density, is used to characterize the nature of the chemical bond in transition-metal hydride and dihydrogen complexes.

1 $$\mathrm{LOL}(r)=\frac{\tau ({\rm{r}})}{1+\tau ({\rm{r}})},\,\tau ({\rm{r}})=\frac{{D}_{0}({\rm{r}})}{(1/2)\sum {\eta }_{i}|\nabla {\varphi }_{i}{({\rm{r}})}^{2}}$$

where *η*_i_ is occupation number of orbital *i*, *φ* is orbital wavefunction. D_0_(r) can be considered as Thomas-Fermi kinetic energy density. Localized orbital locator (LOL) were obtained by employing the Multiwfn software version 3.3.6^43^. The linear optic property was reflected by the isotropic average polarizability (*α*_tot_) calculated as:^44^

2 $$\alpha =\frac{1}{3}({\alpha }_{xx}+{\alpha }_{yy}+{\alpha }_{zz})$$

The third-order NLO properties of the complexes were measured by the second hyperpolarizability (*γ*_tot_), which has been calculated using the following expression:^45^

3 $${\gamma }_{tot}=\frac{1}{5}({\gamma }_{xxxx}+{\gamma }_{yyyy}+{\gamma }_{zzzz}+2{\gamma }_{xxyy}+2{\gamma }_{xxzz}+2{\gamma }_{yyzz})$$

Three methods CAM-B3LYP, BHandHLYP and M06-2X have been employed to ensure the reliability of the DFT approach for the evaluation of electric response properties, and in particular for the estimation of hyperpolarizabilities. The *γ*_tot_ trends of three methods are consistent with each other. Among them, the CAM-B3LYP has been employed with success in prediction of (hyper)polarizabilities^46, 47^. Specifically, the *α* and *γ* values of CAM-B3LYP reproduce the corresponding CCSD(T) figures with 1% and 2%, respectively^46^. Therefore, the results of CAM-B3LYP will be discussed in detail.

## Results and Discussion

### The bonding property and orbital energy level

**Figure 2:**
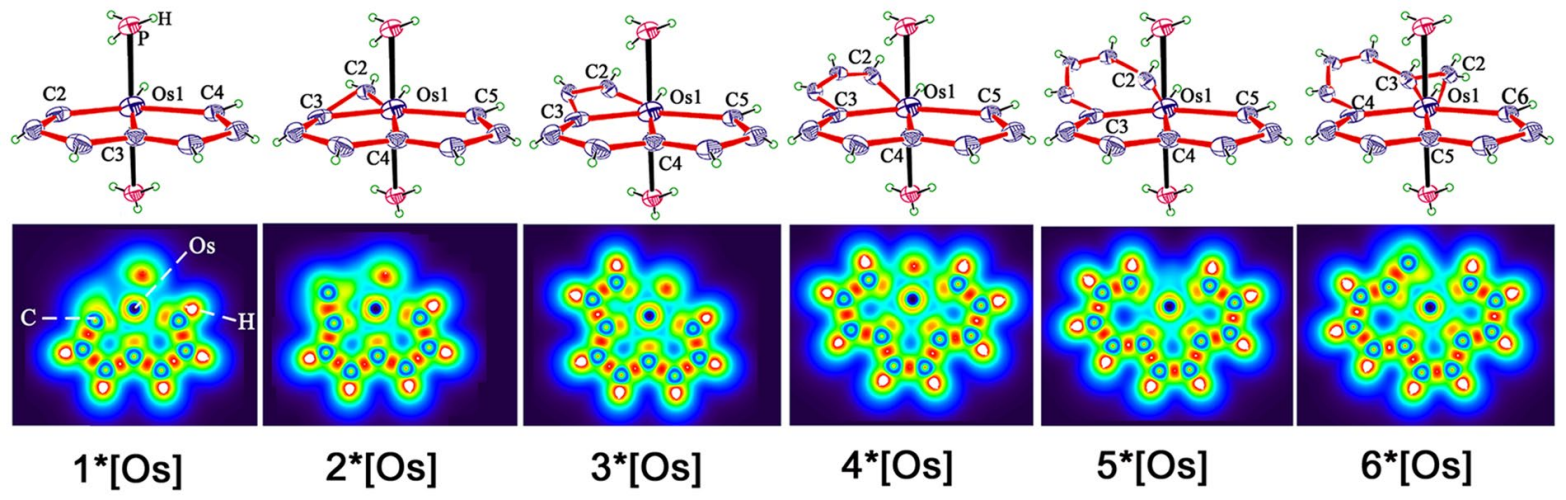
The structures and Localized orbital locator diagrams (LOL) of the complexes 1–6*[Os]: the cut–plane (LOL values from 0.0 to 0.8) is along the y axis with a distance of 0.0 Å from the xz plane. Localized electrons was shown in yellow–red (LOL > 0.5); The pale green basins (LOL ≈ 0.5) characterize delocalized electrons; the electron depletion regions are shown by the blue circles (0.0 < LOL < 0.5).

To elucidate the bonding and electronic structures of these unique pentadentate carbon chain chelates, the structure optimization was performed on B3LYP/def2-TZVPP level of theory (Fig. 2). It estimates the geometric parameters like bond angle and length (Table 1, S1, S2, S3 and S4). Complexes 1–6*[M] (M = Fe, Re, Os and Ir) are essentially planar metal bridged aromatic molecules because the dihedral angles between different rings are ranging from 179.5° to 180.0° (Figs 2, S1, S2 and S3). The C–C bond lengths (1.37–1.42 Å) are similar to those of benzene (1.40 Å), suggesting the aromatic π-conjugation. Results in Table 1 show that the calculated values of bond length in 1–6*[Os] were well coincident with literature values (Table S1).

**Table 1:** AIM parameters, electron density (*ρ*(r)), Laplacian ∇^2^ *ρ*(r), density of the total energy of the electron (*H*(r)), kinetic electron energy density (*G*(r)), potential electron energy density (*V*(r)) for LCPs of the complexes and the delocalization index (DI).

Complex	bond	*ρ*(r)	∇^2^ *ρ*(r)	*G*(r)	*V*(r)	*H*(r)	DI
1*[Os]	Os1–C2	0.1972	0.2130	0.1759	−0.2985	−0.1227	1.9656
	Os1–C3	0.1095	0.1683	0.0843	−0.1264	−0.0418	1.0047
	Os1–C4	0.1275	0.1791	0.0985	−0.1522	−0.0537	1.1713
2*[Os]	Os1–C2	0.0925	0.1325	0.0705	−0.1272	−0.0431	0.8826
	Os1–C3	0.1472	0.1657	0.1148	−0.1881	−0.0733	1.1827
	Os1–C4	0.1154	0.1828	0.0914	−0.1370	−0.0457	1.0344
	Os1–C5	0.1312	0.2095	0.1082	−0.1640	−0.0558	1.2257
3*[Os]	Os1–C2	0.1079	0.1232	0.0724	−0.1141	−0.0416	1.0541
	Os1–C3	0.1330	0.1537	0.0979	−0.1574	−0.0595	1.1117
	Os1–C4	0.1198	0.1526	0.0870	−0.1359	−0.0489	1.0516
	Os1–C5	0.1208	0.1710	0.0920	−0.1412	−0.0492	1.1590
4*[Os]	Os1–C2	0.1177	0.1471	0.0843	−0.1318	−0.0475	0.5195
	Os1–C3	0.1175	0.1580	0.0867	−0.1340	−0.0473	0.5181
	Os1–C4	0.1175	0.1580	0.0867	−0.1340	−0.0472	0.5621
	Os1–C5	0.1176	0.1468	0.0841	−0.1316	−0.0475	0.5633
5*[Os]	Os1–C2	0.1237	0.2004	0.1009	−0.1518	−0.0508	0.5951
	Os1–C3	0.1251	0.1904	0.0993	−0.1511	−0.0518	0.5804
	Os1–C4	0.1119	0.1573	0.0832	−0.1271	−0.0439	0.4976
	Os1–C5	0.1275	0.1674	0.0964	−0.1510	−0.0546	0.6037
6*[Os]	Os1–C2	0.0950	0.1678	0.0744	−0.1069	−0.0325	0.9256
	Os1–C3	0.1273	0.1695	0.0986	−0.1548	−0.0562	1.0667
	Os1–C4	0.1207	0.1849	0.0950	−0.1437	−0.0487	1.1458
	Os1–C5	0.1157	0.1543	0.0851	−0.1315	−0.0465	1.0028
	Os1–C6	0.1230	0.1688	0.0932	−0.1443	−0.0511	1.1605

**Figure 3:**
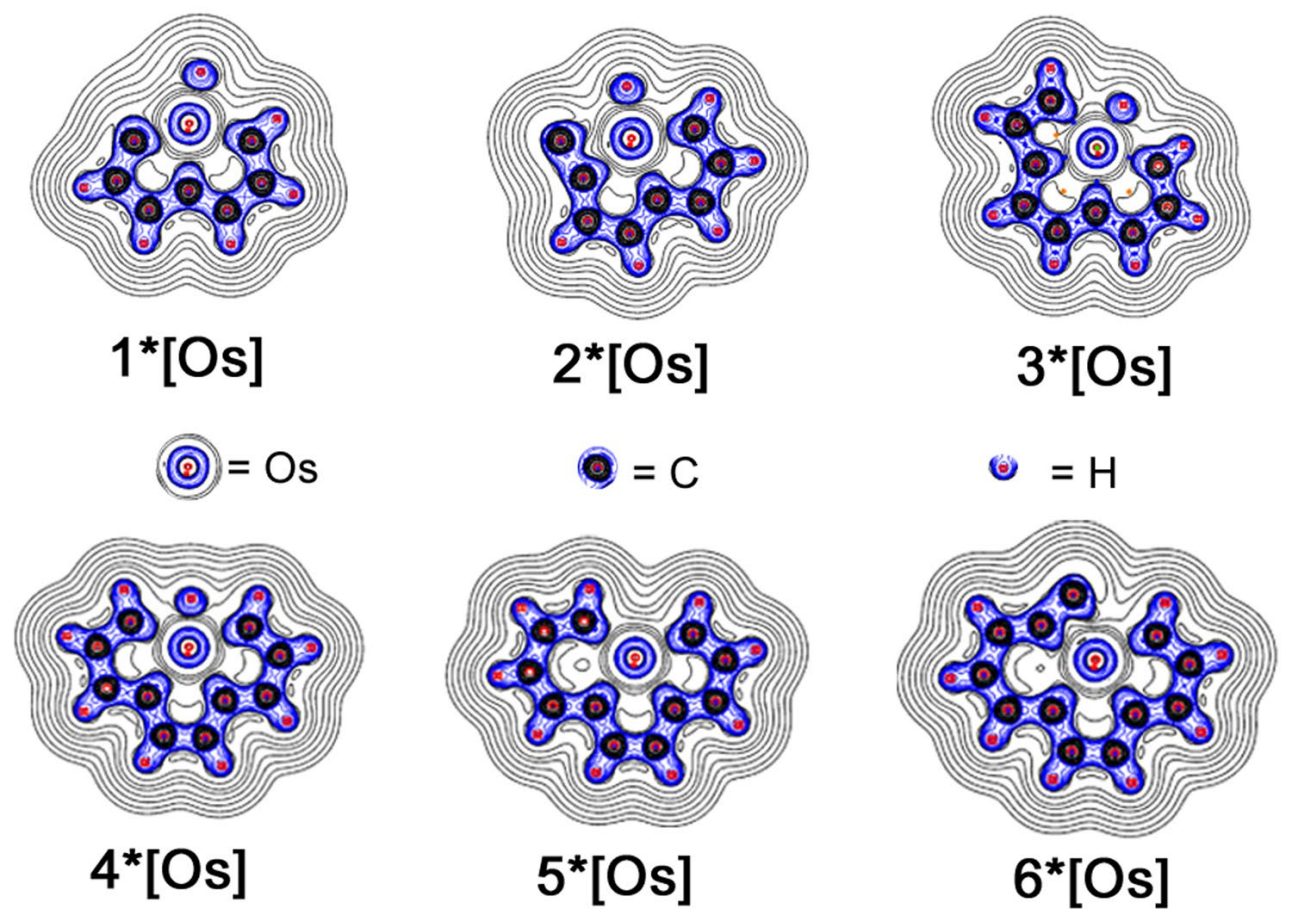
Deformation of electron density distribution of the complexes. (Black and blue lines represent the increase and decrease density areas).

The LOL of the complexes were drawn to interpret the chemical bond in a clear and intuitive manner. The light red and yellow regains between Os and C indicate that covalent bonds are formed, however, these bonds are relatively weaker when compared to the strong covalent C–C bond as depicted in red regains (Fig. 2). In order to qualitatively and quantitatively study the bond strength, bond order (Tables S2–S4) were also taken into account. The calculated wiberg bond indices (WBI) for the Os–C bonds in 1*Os are 1.78, 0.73 and 0.91 for Os1≡C2, Os1–C3, and Os1–C4, respectively, indicating little covalent Os–C bonding characters between the osmium center and these carbons. On the other hand, the bonding characters of the Os–C bonds are clearly shown by the deformation map of the electron density distribution^48^. Deformation map of electron density shows the electron density variation during the formation of a molecule, which is actually molecular electron density minus electron densities of each atom in free-state. As expected, a significant amount of electron density concentrates towards C–C bonding regions that correspond to typical covalent bonds. For covalent bonds, the local energy density at the site of maximum concentration of electron density is always negative (blue line), however, for ionic bonds, H bonds, or Van der Waals bonds, electron density are always positive (black line). The ionic character of the Os–C bonds is observed (Fig. 3).

In order to further determine the bonding type, the Quantum Theory of Atoms (QTAIM)^49^ rigorously provides the nature of the interaction between two atoms (whether covalent, coordinate, and/or ionic) by electron density *ρ*(r) topologies and its derivatives in the line critical point (LCP)^50^. As for the ∇^2^ *ρ*(r) value, the negative value of ∇^2^ *ρ*(r) indicates the shared electron (covalent) interactions. However, a positive value of ∇^2^ *ρ*(r) means the closed-shell interactions. Therefore, the bond between Os and C was not pure covalent bond. The evaluation of the local kinetic energy density, *G*(r), and the local potential energy density *V*(r) have also been listed in Table 1. The values of the total electron energy density (*H*), *H*(r)* = G*(r) + *V*(r), *V*(r) < 0 by definition and *G*(r) > 0 also by definition, reveal the characteristics of the interactions. One can also use the ratio |*V*(r)|/*G*(r) as another useful description; |*V*(r)|/*G*(r) < 1 is characteristic of a typical ionic interaction and |*V*(r)|/*G*(r) > 2 is defined as a “classical” covalent interaction. Taking all these criteria into consideration, the topological properties at LCPs indicate a mixed (partially ionic and partially covalent) character of these coordination bonds because of 1 < |*V*(r)|/*G*(r) < 2^51^. As shown in Table 1, for all complexes, the Os-C bonding interactions are characteristic of partially ionic and partially covalent owing to 1 < |*V*(r)|/*G*(r) < 2. The delocalization index (DI)^52^ measures the number of shared electrons between two atoms and is unity for an equally shared pair of electrons. It is correlated to bond order listed in Table S1.

The energies of the highest occupied molecular orbitals (HOMOs) and the lowest unoccupied molecular orbitals (LUMOs) of 1–6*[M] were shown in Figures S4–S7. The energy gaps between HOMO and LUMO indicate a lowest transition energy for electrons to be excited from the ground state to the excited state. The energy gap of 1*[Os] and 2*[Os] are calculated to be 3.81 and 3.61 eV, much larger than that of 3–6*[Os]. Similarly, the energy gaps of 3*[Os] and 4*[Os] were shown larger values with respect to that of 5*[Os] and 6*[Os]. It appears that incremental conjugate rings greatly decreases the energy gaps between HOMO and LUMO. It is generally believed that low-lying energy gap might be helpful for enhancing the NLO responses of the complexes. Therefore, we can predict that 5*[Os] and 6*[Os] possess relatively larger NLO responses and were expected to be excellent NLO materials.

### Aromatic properties

**Figure 4:**
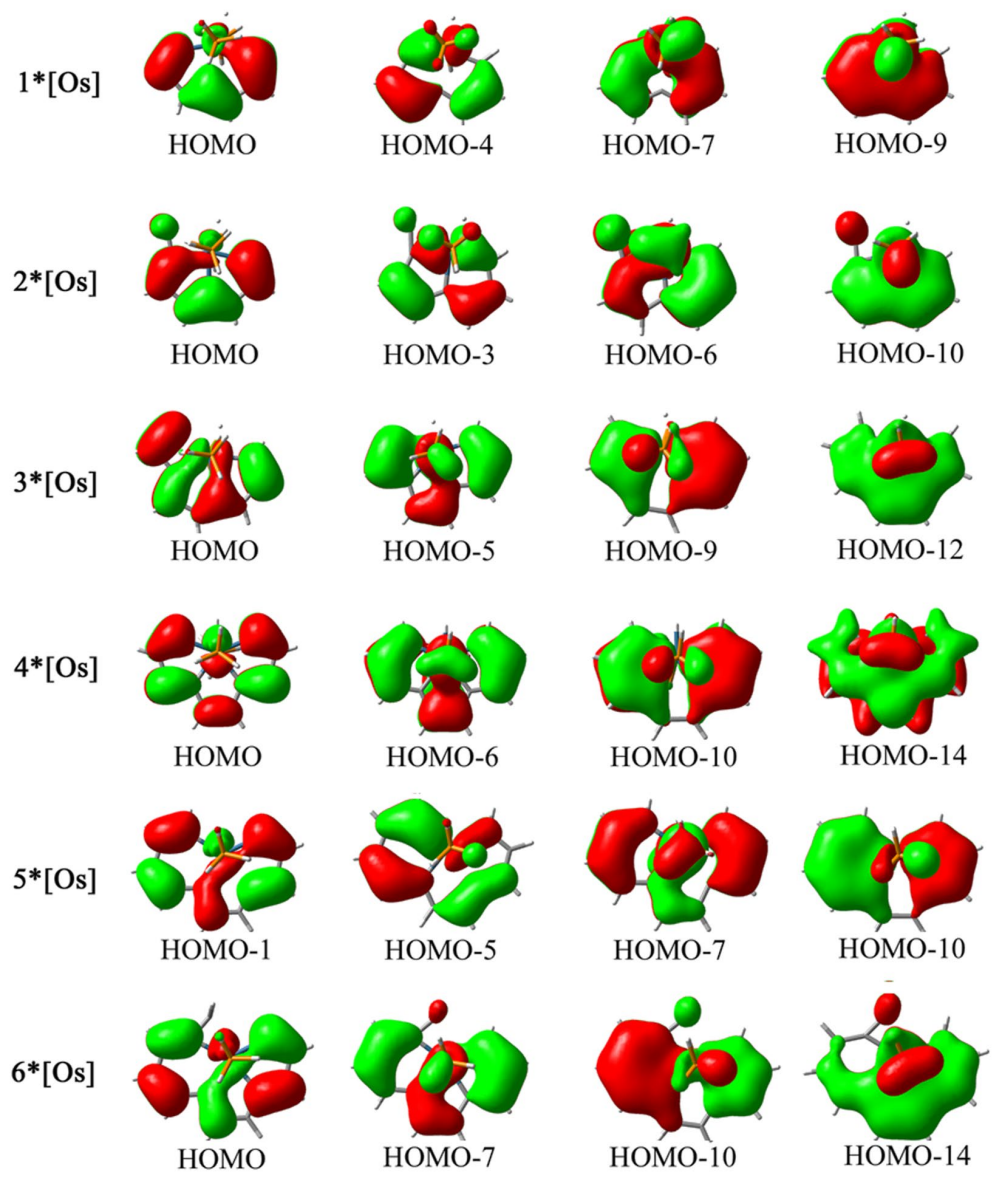
Pictures of selected canonical molecular orbitals (CMOs) calculated at the B3LYP/def2-TZVPP level.

For the sake of exploring the nature of the chemical bonding and aromaticity in these complexes, we show here that a qualitative bonding analysis of the canonical molecular orbitals (CMOs) for the complexes (Fig. 4), which is the most fundamental tool in elucidating aromaticity of a molecular system. It shows that the planar metallacycle 1*[Os] has extended Möbius aromaticity arising from 8–center–8–electron (8c–8e) d_π_–p_π_ π–conjugation which is in conformity with the literature results^26^. In this complex, the 5–coordinated Os center possesses three electrons in its two unhybridized *d*_π_ atomic orbitals (AOs). The 7–center carbon chain ligand contributes 7 unpaired electrons^9^. A distribution of electrons among metal fragment and carbon chain is only a formal analogy and not without ambiguity. These π–AOs constitute seven occupied π molecular orbitals (MOs) of 1*[Os]. In the same way, 2*[Os] have extended Möbius aromaticity. The delocalized plane carbon–carbon single bond could be attributed to the σ–aromaticity and π–aromaticity are shown in its three–membered and five–membered rings, respectively^27^. For 3*[Os], π orbital diagram of HOMO-12 reveals the 10c–10e π–conjugation. The metal fragment decreases the antiaromaticity in cyclobutadiene and pentalene simultaneously. The four membered ring is antiaromaticity while two fused five-membered rings are aromaticity. The aromaticity of 4*[Os] arise from three aromatous five-membered rings. For 5*[Os], the aromaticity arising from 12c–12e architecture. Similar to 5*[Os], 6*[Os] shows that the five coordinated carbons lie in the equatorial plane, representing the highest carbon coordination number for a metal atom in a planar geometry^30^.

**Figure 5:**
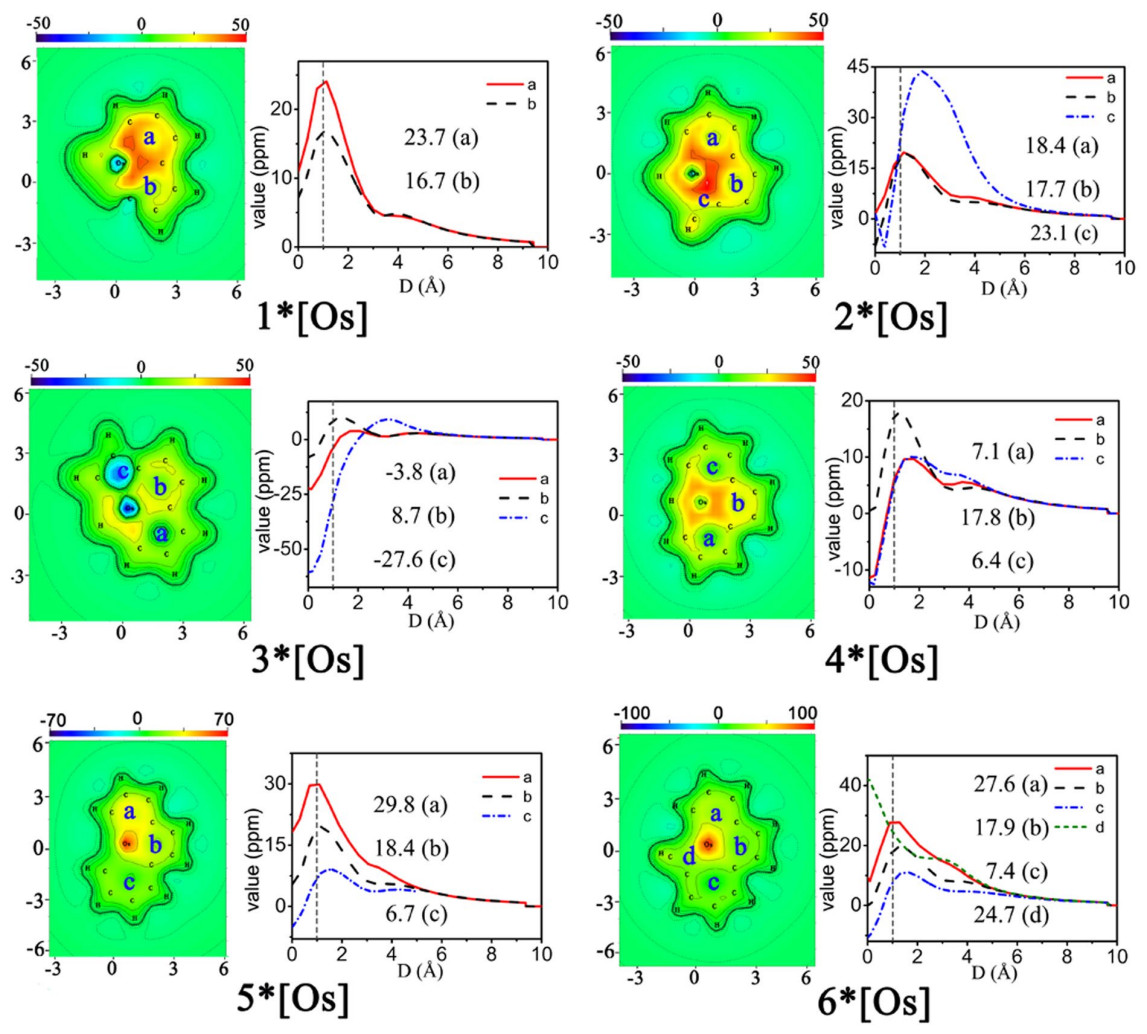
The cut–plane map of iso–chemical shielding surface ICSS_zz_ and scanning line of –NICS_ZZ_ values for 1–6*[Os].

**Figure 6:**
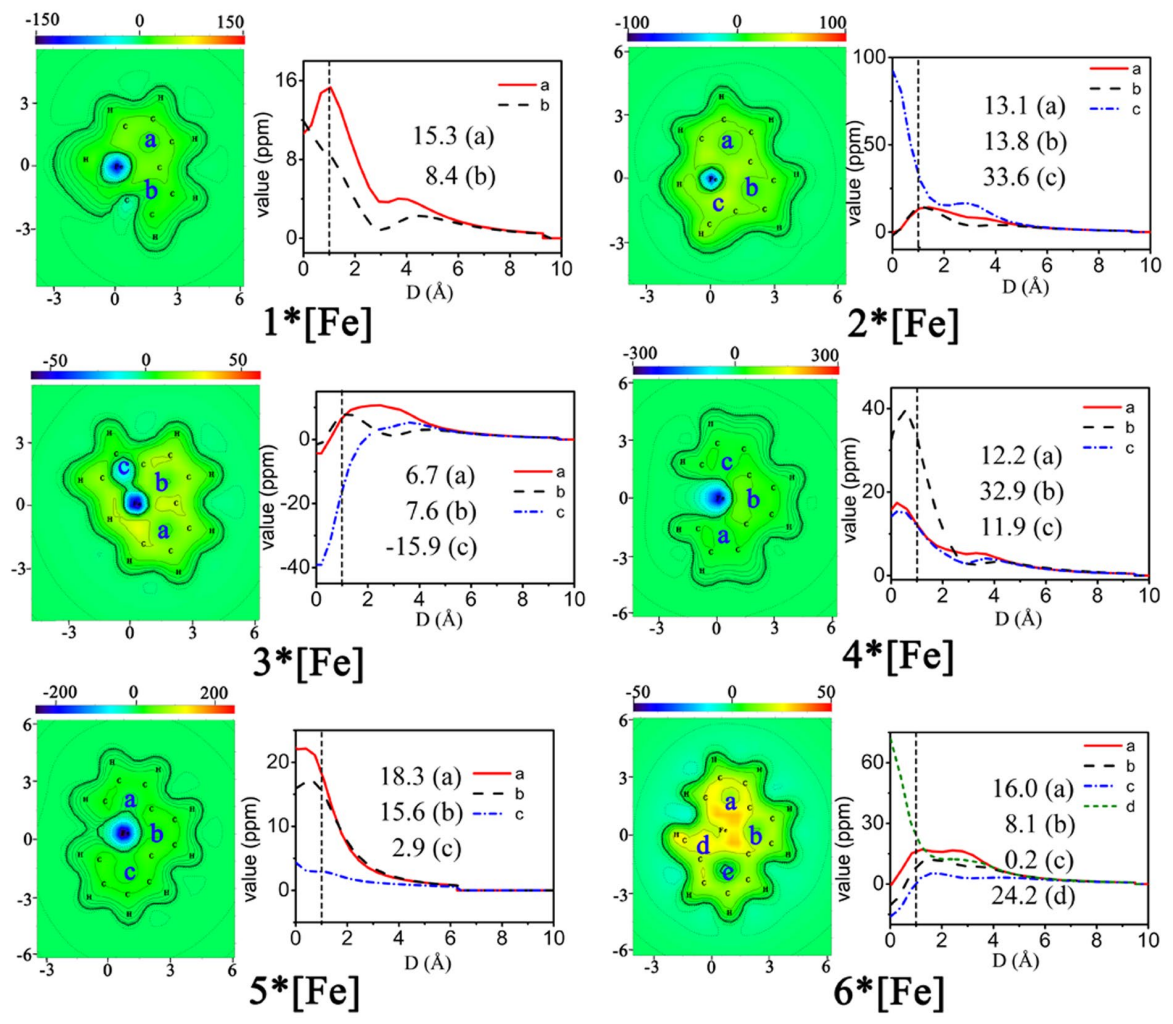
The cut–plane map of iso–chemical shielding surface ICSS_zz_ and scanning line of –NICS_ZZ_ values for 1–6*[Fe].

Complementary analyses (such as NICS calculations and other aromaticity indexes) offer additional or independent support for the assessment of CMO analysis. It is emphasized here that the CMO analyses and electron counting are the most fundamental tools in elucidating aromaticity. Complementary analyses (such as NICS calculations) only offer additional or independent support for the assessment. Since NICS as a criterion of aromaticity has been documented to fail in a number of cases (in particular in metal clusters)^53–56^, we are inclined to state that the aromaticity of these complexes on account of negative NICS values. With the aim of quantitatively evaluating the aromatic character of these novel metal-bridged aromatic systems, the nucleus-independent chemical shift (NICS) values were calculated. NICS is commonly studied at some special points (e.g. ring center), and can be investigated by scanning its value in a line (1D) or in a plane (2D). On the other hand, the so-called iso-chemical shielding surface (ICSS) actually is the isosurface of NICS, which clearly exhibits the distribution of NICS and thus presents a very intuitive picture on aromaticity. Present function is used to generate grid data and visualize isotropic ICSS, anisotropic ICSS, ICSS_XX_, ICSS_YY_ and ICSS_ZZ_, they correspond to the isosurface of NICS, NICS_ani_, NICS_XX_, NICS_YY_ and NICS_ZZ_, respectively. For planar systems, the component form of ICSS must be more meaningful and useful than ICSS, just like NICS_ZZ_ has conspicuous advantage over NICS. Thus, we chose to generate ICSS_ZZ_ for the purpose of NICS_ZZ_. The cut-plane map representations are perpendicular to the z axis with a distance of 1 Å from the *xy* plane, which represent NICS(1)_ZZ_ (Figs 5, 6, S8 and S9). Aromaticity is colored by green–yellow–red, while antiaromaticity is shown in cyan–light blue–dark blue. The line charts present the variation of –NICS(1)_ZZ_ values with increasing perpendicular distance from xy plane, specifically, the ring centers.

In general, negative values of NICS(1)_zz_ indicate aromaticity and positive values are antiaromaticity. The NICS(1)_zz_ values at the centres of rings *a* and *b* in 1*[Os] are −23.7 and −16.7 ppm, respectively, which is in keeping with the cut–plane which shows that aromaticity of ring *a* is more obvious (Fig. 5). The NICS(1)_zz_ value of ring *c* (−23.1 ppm) for 2*[Os] are visually smaller than ring *a* (−18.4 ppm) and ring *b* (−17.7 ppm). For 3*[Os], ring *a* and *c* are filled with light blue indicating its antiaromaticity, and the NICS(1)_zz_ value of ring *a* and *c* is 3.8 and 27.6 ppm. Ring *b* shows aromaticity (−8.7 ppm). Inspection of 4*[Os] reveals that the NICS(1)_zz_ value of ring *b* (−17.8 ppm) in the middle is significantly smaller with respect to that of ring *a* (−7.1 ppm) and ring *c* (−6.4 ppm), suggesting that aromaticity of the middle ring is more obvious. With regard to 5*[Os], the NICS(1)_zz_ value of ring *a*, *b* and *c* are −29.8, −18.4 and −6.7 ppm, respectively. For 6*[Os], NICS(1)_zz_ value of ring *a*, *b*, *c* and *d* are −27.6, −17.9, −7.4 and −24.7 ppm, respectively, which indicates that two rings at the ends of the complex possess larger aromaticity when compared with two rings in the middle. The NICS values of 5*[Os] and 6*[Os] are smaller than others, indicating that the aromaticities of 5*[Os] and 6*[Os] are larger ones. As shown in Fig. 6, the NICS(1)_zz_ values of rings *a* and *b* in 1*[Fe] are −15.3 and −8.4 ppm, respectively, which are larger with respect to that of 1*[Os]. It indicates that the aromaticity of 1*[Fe] is smaller than that of 1*[Os]. The same result can be achieved from 3*[Fe], 5*[Fe] and 6*[Fe]. Moreover, inspection of Figure S8 reveals that the NICS(1)_zz_ values of complexes 1–6*[Re] are all negative and smaller, declaring that the aromaticity of 1–6*[Re] are larger ones as compared to that of 1–6*[Os]. With regarded to 1–6*[Ir], the NICS(1)zz values of rings *a* and *b* in 1*[Ir] are −9.3 and −10.0 ppm (Figure S9), respectively, larger than that of 1*[Os]. Similar results can be observed in 2–6*[Ir]. Thus, the aromaticities of 1–6*[Ir] are smaller with respect to 1–6*[Os]. A general rule can be obtained, that is, for the metal compounds in the same period, the aromaticity presents a roughly weakening trend with the decrease of metal radius.

To ensure the accuracy of the results, we performed calculations using the quantum theory of atoms-in-molecules (QTAIM). Here, the Multiwfn program is used to generate the para-delocalization index (PDI), the multicenter bond order aromaticity index (MCBD)^57^, Shannon aromaticity index (SA)^58^ and curvature of electron density^59^ of 1–6*[Os]. The PDI, MCBD, SA and curvature datas for 1–6*[Os] are presented in Table S7. Larger PDI and MCBO value corresponds to stronger aromaticity. The smaller the SA index, the more aromatic is the molecule. The range of 0.003 < SA < 0.005 is chosen as the boundary of aromaticity/antiaromaticity in original paper. The more negative the curvature, the stronger the aromaticity. The results of these aromaticity indexes verify the results of NICS and ICSS on the side.

### UV-vis absorption spectrum and electron transition

**Figure 7:**
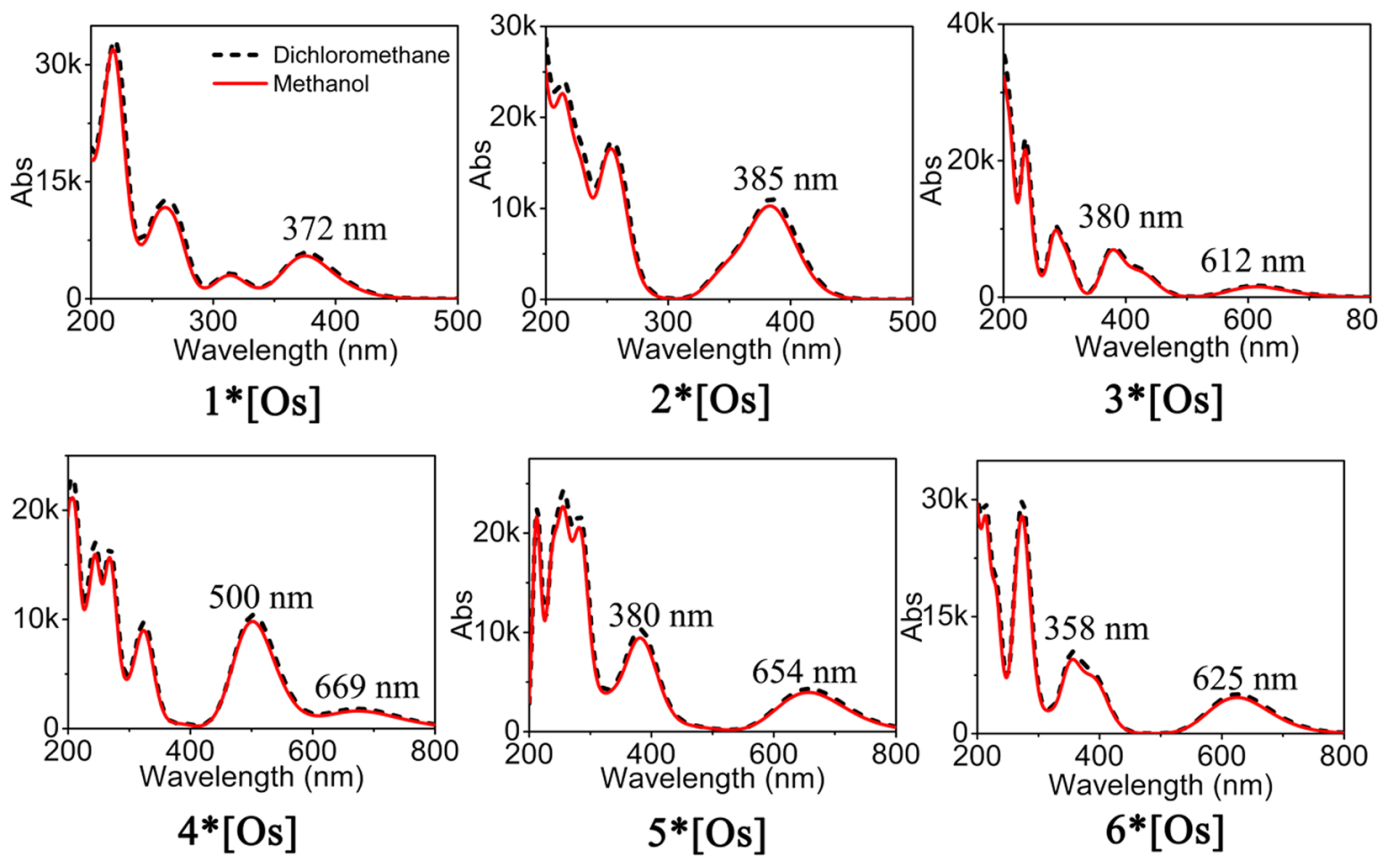
Absorption spectra (Abs: A is absorbance coefficient, L/(g·cm). b is the layer thickness, cm. c is solution concentration, g/L.) of the complexes 1–6*[Os] in the solvents of dichloromethane and methanol obtained at B3LYP/def2-TZVPP.

**Figure 8:**
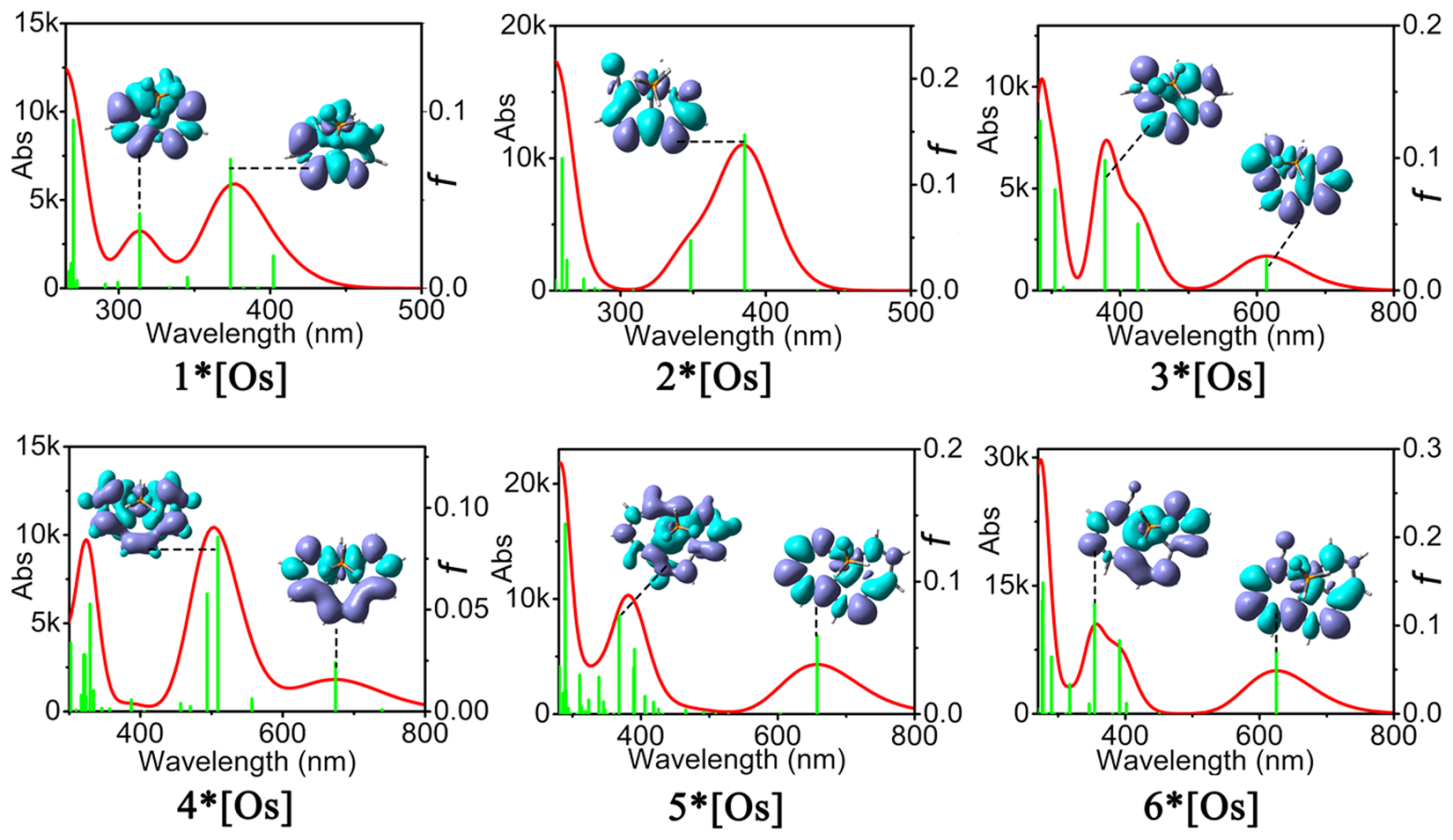
Absorption spectra of the complexes 1–6*[Os] along with electron density difference maps, in which purple and blue colors indicate accumulation and depletion of electron density, respectively, obtained at B3LYP/def2-TZVPP.

The absorption spectra of these complexes in two solvents show that the spectra in dichloromethane and methanol are exactly similar (Figs 7 and S10–S12), thus we choose the former of 1–6*[Os] to study the absorption spectra (Fig. 8). The absorption maximum of complex 2*[Os] in the low–energy absorption band located at 385 nm, which is a little red–shifted by 13 nm compared with that of 1*[Os] (*λ*_max_ = 372 nm) due to that the additionally coordinate C2 atom in 2*[Os] was not involved in the effective conjugation of surface. Remarkably, the low–energy absorption band (*λ*_max_ = 612 nm) of 3*[Os] is red–shifted by 227 nm with respect to that of 1*[Os], which results from the increased delocalized π electrons and the enlargement of conjugate surface. Similarly, the low–energy absorption band (*λ*_max_ = 669 nm) of 4*[Os] is largely red–shifted by 297 nm when compared with that of 1*[Os] because of the increased delocalized electrons. The low–energy absorption band of 5*[Os] (*λ*_max_ = 654 nm and 6*[Os] (*λ*_max_ = 625 nm) are also red–shifted by 282 and 253 nm compared with that of 1*[Os]. Next, conducting a comparative study of absorption intensities and oscillator strengths of 3*[Os], 4*[Os], 5*[Os] and 6*[Os] appear particularly important. Inspection of the spectra reveals that the absorption intensities of these four complexes are 1615, 1890, 4229 and 4861, respectively. And their oscillator strengths are 0.0233, 0.0253, 0.0581 and 0.0671, respectively, showing that along with the increased conjugate surface, low–energy absorption band was red–shifted and absorption intensity as well as oscillator strength were increased. As shown in the electron density difference maps, there are unconspicuous intramolecular charge transfer (CT), including CT from metal to ligand, ligand to metal, or intramolecular CT within ligands. And, of course, they also have a small amount of π–π* of electron transition, which is the main reason for the organic molecules with large NLO response.

### The liner and nonlinear optical properties

**Figure 9:**
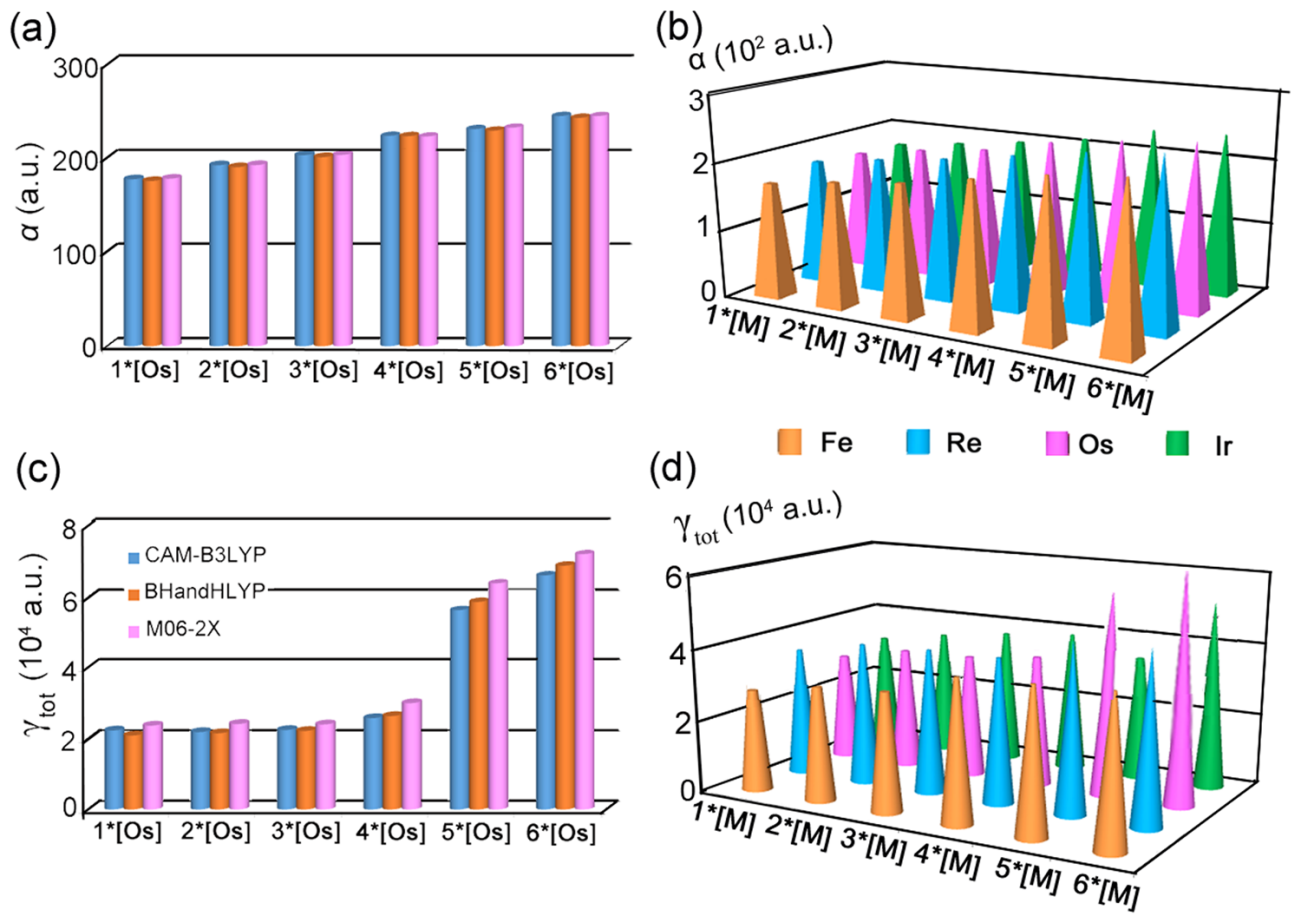
(**a**) α values (a.u.) of 1–6*[Os] at three methods with def2-TZVPP basis set; (**b**) *γ*_tot_ values (10^4^ a.u.) of 1–6*[Os] at three methods with def2-TZVPP basis set; (**c**) α values (10^2^ a.u.) of 1–6*[M] obtained at B3LYP/def2-TZVPP. **(d**) *γ*_tot_ values (10^4^ a.u.) of 1–6*[M] obtained at B3LYP/def2-TZVPP.

In order to guarantee the reliability of the results, three methods have been used to calculated the α values. The results (Fig. 9a) show that the α values are not sensitive to methods. The results of CAM-B3LYP were chosen to discuss in detail. The liner optical response data shows that the orders of *α* values for these four kinds of metal organic complexes are semblable (Fig. 9b). Taking 1–6*[Os] as examples, the order of *α* values follows as 6*[Os] (2.45 × 10^2^ a.u.) > 5*[Os] (2.31 × 10^2^ a.u.) > 4*[Os] (2.24 × 10^2^ a.u.) > 3*[Os] (2.03 × 10^2^ a.u.) > 2*[Os] (1.92 × 10^2^ a.u.) > 1*[Os] (1.78 × 10^3^ a.u.), indicating that the *α* values increase with the increasing number of conjugated C atoms. It is noteworthy that *α* values of different kinds of metal organic complexes increase as 6*[Fe] (2.37 × 10^2^ a.u.) < 6*[Ir] (2.39 × 10^2^ a.u.) < 6*[Os] (2.47 × 10^2^ a.u.) < 6*[Re] (2.51 × 10^2^ a.u.) shown in Tables S8–S11. In order to provide an original understanding of the *α* values, we focused on the relative electronic spatial extent 〈R^2^〉. To the best of our knowledge, the 〈R^2^〉 is a physical property that characterizes the electron density volume around the molecule^60^. The 〈R^2^〉 values in series increase as 6*[Os] (3.12 × 10^3^ a.u.) > 5*[Os] (2.92 × 10^3^ a.u.) > 4*[Os] (2.54 × 10^3^ a.u.) > 3*[Os] (2.20 × 10^3^ a.u.) > 2*[Os] (2.16 × 10^3^ a.u.) > 1*[Os] (1.98 × 10^3^ a.u.), which is in well accordance with the decreasing order of the *α* values.

The third–order NLO properties of the complexes were measured by the second hyperpolarizability (*γ*_tot_). As is well known that the γ values are sensitive to the methods, thus, CAM-B3LYP, BHandHLYP and M06-2X have been employed. From the Fig. 9c, the *γ*_tot_ trends of three methods are consistent with each other. The CAM-B3LYP has been employed with success in prediction of (hyper)polarizabilities. Therefore, the results of CAM-B3LYP were chosen to discuss following. The orders of *γ*_tot_ values for these four kinds of metal organic complexes are also semblable (Fig. 9d). Taking 1–6*[Os] as examples, the *γ*_tot_ value of 6*[Os] (6.62 × 10^4^ a.u.) is evaluated to be largest and 1*[Os] possess the smallest value of *γ*_tot_ (2.13 × 10^4^ a.u.), which is in conformity with previous prediction by means of *E*_gap_. The decreasing trend of *γ*_tot_ values for these complexes can be concluded by further observation of Fig. 8c, that is 6*[Os] > 5*[Os] (5.64 × 10^4^ a.u.) > 4*[Os] (2.58 × 10^4^ a.u.) > 3*[Os] (2.25 × 10^4^ a.u.) > 2*[Os] (2.19 × 10^4^ a.u.) > 1*[Os]. 5*[Os] and 6*[Os] possess considerab γ_tot_ values with respect to that of others. Combining with the above conclusion of the aromaticity, a novel relation between aromaticity and third order NLO response can be created that the metal–bridged polycyclic complex with better aromaticity is expected to exhibit larger third order NLO responses. To have an insight into the origin of third–order NLO responses, two–level model of the *γ*_tot_ is considered for the studied complexes, which is the linkage between the *γ*_tot_ and electronic transition(s) in low–lying crucial excited states. Therefore, using the two–level models^61^ is a reliable way to analyze second hyperpolarizabilities and the expression is as follows: *f* ^2^/*E* ^5^. where *f* the oscillator strength, and *E* the transition energy. In the two–level expression, the *γ*_tot_ value is proportional to the quadratic power of *f* but inversely proportional to the fifth power of *E*. *f* ^2^/*E* ^5^ of low–lying crucial excited states have been calculated as the order: 6*[Os] (1.58 × 10^−4^) > 5*[Os] (1.56 × 10^−4^) > 4*[Os] (2.74 × 10^−5^) > 3*[Os] (1.57 × 10^−5^) > 2*[Os] (1.38 × 10^−5^) > 1*[Os] (1.33 × 10^−5^), which is in quantitative agreement with the order of *γ*_tot_ values (Table S16). Particularly noteworthy is that the *γ*_tot_ values of different kinds of metal organic complexes increase as 6*[Fe] (2.50 × 10^4^ a.u.) < 6*[Re] (3.39 × 10^4^ a.u.) < 6*[Ir] (5.62 × 10^4^ a.u.) < 6*[Os] (6.62 × 10^4^ a.u.).

## Conclusions

Overall, for metal–bridged polcyclic complex, the aromaticity was attributed to Craig–Möbius topology which favours 4n π–electron conjugation counts. The introduction of a metal fragment is an efficient strategy to stabilize cyclic alkynes by reducing ring strain. The current study showed the atomic radius of center metal greatly influence the bond, aromaticity. Specifically, smaller metal radius is conducive to the formation of the bond. With regard to the metal compounds in the same period, the aromaticity decreases and might even become the antiaromaticity as the decrease of metal radius. It reveals useful information for scientists to develop new applications of metal regulatory mechanism. The *α* values increase with the increasing number of conjugated C atoms, which is in well accordance with the increasing order of the electronic spatial extent values. Significantly, a novel relationship between aromaticity and third order NLO response can be concluded that the metal–bridged polycyclic complex with better aromaticity will exhibit larger third order NLO responses, which opens new perspectives to discriminate and design improved NLO materials.

## Electronic supplementary material

Supporting information
