# Supplementary material for: The novel link between planar möbius aromatic and third order nonlinear optical properties of metal–bridged polycyclic complexes
Source: Sci Rep. 2017 Aug 31;7:10182. doi: 10.1038/s41598-017-10739-7 (PMC5579294; doi:10.1038/s41598-017-10739-7)
Supplement: Supplementary file 1 — Supporting information [file 41598_2017_10739_MOESM1_ESM.doc]

**The novel link between planar möbius aromatic and third order nonlinear optical properties of metal–bridged polycyclic complexes**

**Li Wang,^1^ Jinting Ye,^1^ Hongqiang Wang,^1^ Haiming Xie^1, 2^ & Yongqing Qiu*^1, 2^**

^1^Institute of Functional Material Chemistry, Faculty of Chemistry, Northeast Normal University, Changchun 130024, China.

^2^National & Local United Engineering Laboratory for Power Battery, Faculty of Chemistry, Northeast Normal University, Changchun 130024, China.

* [qiuyq466@nenu.edu.cn](mailto:qiuyq466@nenu.edu.cn) (Y. Q. Qiu)

**Table S1** The experimental and calculated results of bond lengths (L, Å) and wiberg bond order (WBI) between Os and C in complexes **1–6*[Os]** at B3LYP/def2-TZVPP.

| Complex | parameter | Os1–C2 | Os1–C3 | Os1–C4 | Os1–C5 | Os1–C6 |
| --- | --- | --- | --- | --- | --- | --- |
| **1*[Os]** | L (exp) | 1.85 | 2.10 | 2.06 |  |  |
|  | L (cal) | 1.85 | 2.16 | 2.08 |  |  |
|  | WBI | 1.78 | 0.73 | 0.91 |  |  |
| **2*[Os]** | L (exp) | 2.27 | 2.03 | 2.08 | 2.08 |  |
|  | L (cal) | 2.27 | 2.00 | 2.13 | 2.06 |  |
|  | WBI | 0.60 | 0.88 | 0.76 | 0.96 |  |
| **3*[Os]** | L (exp) | 2.17 | 2.09 | 2.11 | 2.11 |  |
|  | L (cal) | 2.17 | 2.07 | 2.12 | 2.11 |  |
|  | WBI | 0.80 | 0.88 | 0.79 | 0.89 |  |
| **4*[Os]** | L (exp) | 2.15 | 2.07 | 2.15 | 2.12 |  |
|  | L (cal) | 2.13 | 2.13 | 2.13 | 2.13 |  |
|  | WBI | 0.86 | 0.80 | 0.80 | 0.85 |  |
| **5*[Os]** | L (exp) | 2.06 | 2.11 | 2.14 | 2.10 |  |
|  | L (cal) | 2.09 | 2.09 | 2.16 | 2.09 |  |
|  | WBI | 0.85 | 0.88 | 0.71 | 0.91 |  |
| **6*[Os]** | L (exp) | 2.25 | 2.03 | 2.10 | 2.11 | 2.09 |
|  | L (cal) | 2.21 | 2.06 | 2.11 | 2.14 | 2.10 |
|  | WBI | 0.62 | 0.73 | 0.87 | 0.73 | 0.89 |

**Table S2.** The calculated results of bond lengths (L, Å), wiberg bond order (WBI) and bond energy (*E*_bond_, kJ/mol) between Fe and C in complexes **1-6*[Fe]**.

| Complex |  | Fe1-C2 | Fe1-C3 | Fe1-C4 | Fe1-C5 | Fe1-C6 |
| --- | --- | --- | --- | --- | --- | --- |
| **1*[Fe]** | L | 1.70 | 2.06 | 1.96 |  |  |
|  | WBI | 1.49 | 0.57 | 0.81 | WBI |  |
| **2*[Fe]** | L | 2.13 | 1.90 | 2.02 | 1.94 |  |
|  | WBI | 0.46 | 0.63 | 0.64 | 0.85 |  |
| **3*[Fe]** | L | 2.03 | 1.94 | 2.00 | 2.01 |  |
|  | WBI | 0.68 | 0.73 | 0.66 | 0.70 |  |
| **4*[Fe]** | L | 1.92 | 1.99 | 1.99 | 1.92 |  |
|  | WBI | 0.89 | 0.71 | 0.71 | 0.89 |  |
| **5*[Fe]** | L | 1.98 | 1.98 | 2.02 | 2.00 |  |
|  | WBI | 0.71 | 0.71 | 0.61 | 0.73 |  |
| **6*[Fe]** | L | 2.12 | 2.00 | 2.02 | 2.02 | 1.99 |
|  | WBI | 0.45 | 0.49 | 0.75 | 0.67 | 0.79 |

**Table S3.** The calculated results of bond lengths (L, Å), wiberg bond order (WBI) and bond energy (*E*_bond_, kJ/mol) between Fe and C in complexes **1-6*[Re]**.

| Complex |  | Re1-C2 | Re1-C3 | Re1-C4 | Re1-C5 | Re1-C6 |
| --- | --- | --- | --- | --- | --- | --- |
| **1*[Re]** | L | 1.86 | 2.23 | 2.08 |  |  |
|  | WBI | 0.49 | 0.20 | 0.25 |  |  |
| **2*[Re]** | L | 2.32 | 2.00 | 2.18 | 2.07 |  |
|  | WBI | 0.15 | 0.25 | 0.21 | 0.26 |  |
| **3*[Re]** | L | 2.12 | 2.09 | 2.18 | 2.13 |  |
|  | WBI | 0.29 | 0.24 | 0.20 | 0.25 |  |
| **4*[Re]** | L | 2.09 | 2.14 | 2.14 | 2.09 |  |
|  | WBI | 1.11 | 0.87 | 0.87 | 1.11 |  |
| **5*[Re]** | L | 2.00 | 2.13 | 2.20 | 2.07 |  |
|  | WBI | 1.09 | 0.96 | 0.78 | 1.02 |  |
| **6*[Re]** | L | 2.22 | 2.01 | 2.15 | 2.15 | 2.11 |
|  | WBI | 0.17 | 0.26 | 0.22 | 0.23 | 0.27 |

**Table S4.** The calculated results of bond lengths (L, Å), wiberg bond order (WBI) and bond energy (*E*_bond_, kJ/mol) between Fe and C in complexes **1-6*[Ir]**.

| Complex |  | Ir1-C2 | Ir1-C3 | Ir1-C4 | Ir1-C5 | Ir1-C6 |
| --- | --- | --- | --- | --- | --- | --- |
| **1*[Ir]** | L | 1.92 | 2.20 | 2.10 |  |  |
|  | WBI | 1.30 | 0.65 | 0.77 |  |  |
| **2*[Ir]** | L | 2.33 | 2.17 | 2.15 | 2.08 |  |
|  | WBI | 0.46 | 0.48 | 0.69 | 0.78 |  |
| **3*[Ir]** | L | 2.16 | 2.13 | 2.15 | 2.16 |  |
|  | WBI | 0.72 | 0.68 | 0.71 | 0.73 |  |
| **4*[Ir]** | L | 2.16 | 2.15 | 2.15 | 2.16 |  |
|  | WBI | 0.73 | 0.70 | 0.70 | 0.73 |  |
| **5*[Ir]** | L | 2.12 | 2.04 | 2.08 | 2.11 |  |
|  | WBI | 0.76 | 0.82 | 0.69 | 0.80 |  |
| **6*[Ir]** | L | 2.26 | 2.17 | 2.15 | 2.10 | 2.13 |
|  | WBI | 0.49 | 0.49 | 0.70 | 0.71 | 0.74 |

**Table S5. Frontier molecular orbital energy levels and the energy gaps between HOMO and LUMO of 1-6*[Fe] and 1-6*[Re].**

| Basis sets for Fe and Re | Complex | H | L | *E*_gap_ | Complex | H | L | *E*_gap_ |
| --- | --- | --- | --- | --- | --- | --- | --- | --- |
| Lan2dz | **1*[Fe]** | -5.64 | -2.25 | 3.39 | **1*[Re]** | -4.97 | -1.60 | 3.37 |
| Def2TZVPP |  | -5.68 | -2.23 | 3.45 |  | -5.03 | -1.61 | 3.42 |
| Lan2dz | **2*[Fe]** | -5.67 | -2.42 | 3.25 | **2*[Re]** | -4.97 | -1.83 | 3.14 |
| Def2TZVPP |  | -5.70 | -2.41 | 3.29 |  | -5.05 | -1.86 | 3.19 |
| Lan2dz | **3*[Fe]** | -5.30 | -2.48 | 2.82 | **3*[Re]** | -5.10 | -2.19 | 2.91 |
| Def2TZVPP |  | -5.31 | -2.49 | 2.82 |  | -5.12 | -2.23 | 2.89 |
| Lan2dz | **4*[Fe]** | -4.88 | -2.51 | 2.37 | **4*[Re]** | -5.38 | -2.79 | 2.59 |
| Def2TZVPP |  | -5.00 | -2.65 | 2.35 |  | -5.43 | -2.82 | 2.61 |
| Lan2dz | **5*[Fe]** | -5.06 | -2.68 | 2.38 | **5*[Re]** | -4.53 | -1.87 | 2.66 |
| Def2TZVPP |  | -5.07 | -2.68 | 2.39 |  | -4.57 | -1.89 | 2.68 |
| Lan2dz | **6*[Fe]** | -4.97 | -2.56 | 2.41 | **6*[Re]** | -4.76 | -1.94 | 2.82 |
| Def2TZVPP |  | -4.98 | -2.55 | 2.43 |  | -4.79 | -1.96 | 2.83 |

**Table S6. Frontier molecular orbital energy levels and the energy gap between HOMO and LUMO of 1-6*[Os] and 1-6*[Ir].**

| Basis sets for Os and Ir | Complex | H | L | *E*_gap_ | Complex | H | L | *E*_gap_ |
| --- | --- | --- | --- | --- | --- | --- | --- | --- |
| Lan2dz | **1*[Os]** | -5.81 | -2.02 | 3.79 | **1*[Ir]** | -3.75 | -1.78 | 1.97 |
| Def2TZVPP |  | -5.81 | -2.00 | 3.81 |  | -4.04 | -2.03 | 2.01 |
| Lan2dz | **2*[Os]** | -5.66 | -2.07 | 3.59 | **2*[Ir]** | -3.85 | -1.02 | 2.83 |
| Def2TZVPP |  | -5.74 | -2.13 | 3.61 |  | -3.90 | -0.79 | 3.11 |
| Lan2dz | **3*[Os]** | -5.15 | -2.34 | 2.81 | **3*[Ir]** | -3.82 | -1.13 | 2.69 |
| Def2TZVPP |  | -5.21 | -2.39 | 2.82 |  | -3.93 | -1.18 | 2.75 |
| Lan2dz | **4*[Os]** | -4.47 | -1.53 | 2.94 | **4*[Ir]** | -4.40 | -1.25 | 3.15 |
| Def2TZVPP |  | -4.54 | -1.57 | 2.97 |  | -4.44 | -1.28 | 3.16 |
| Lan2dz | **5*[Os]** | -4.59 | -2.23 | 2.36 | **5*[Ir]** | -4.92 | -2.45 | 2.47 |
| Def2TZVPP |  | -4.65 | -2.27 | 2.38 |  | -3.76 | -1.24 | 2.52 |
| Lan2dz | **6*[Os]** | -4.82 | -2.17 | 2.65 | **6*[Ir]** | -3.76 | -1.11 | 2.65 |
| Def2TZVPP |  | -4.88 | -2.21 | 2.67 |  | -3.81 | -1.05 | 2.76 |

**Table S7.** Calculated nucleus independent chemical shift (NICS) values (in ppm), as well as other aromaticity indices Para-delocalization index (PDI), Multi-center bond order (MCBO) and Harmonic oscillator measure of aromaticity (HOMA) of **1-6*[Os]** at the B3LYP/def2-TZVPP level.

|  | Ring | NICS(1)_zz_ | PDI | MCBO | SA | curvature |
| --- | --- | --- | --- | --- | --- | --- |
| C_6_H_6_ |  |  |  |  |  |  |
| **1*[Os]** | a | -23.7 | 0.0322 | 0.0126 | 0.0215 | -0.0385 |
|  | b | -16.7 | 0.0261 | 0.0058 | 0.0263 | -0.0239 |
| **2*[Os]** | a | -18.4 | 0.0260 | 0.0052 | 0.0252 | -0.0255 |
|  | b | -17.7 | 0.0230 | 0.0075 | 0.0251 | -0.0243 |
|  | c | -23.1 | 0.0287 | 0.0174 | 0.0231 | -0.0308 |
| **3*[Os]** | a | 3.8 | 0.0003 | 0.0050 | 0.0327 | -0.0020 |
|  | b | -8.7 | 0.0102 | 0.0081 | 0.0267 | -0.0098 |
|  | c | 27.6 | 0.0001 | -0.0284 | 0.0368 | 0.0089 |
| **4*[Os]** | a | -7.1 | 0.0093 | 0.0080 | 0.0287 | -0.0101 |
|  | b | -17.8 | 0.0213 | 0.0093 | 0.0234 | -0.0245 |
|  | c | -6.4 | 0.0090 | 0.0057 | 0.0299 | -0.0086 |
| **5*[Os]** | a | -29.8 | 0.0456 | 0.0101 | 0.0186 | -0.0405 |
|  | b | -18.4 | 0.0261 | 0.0099 | 0.0246 | -0.0257 |
|  | c | -6.7 | 0.0086 | 0.0023 | 0.0279 | -0.0096 |
| **6*[Os]** | a | -27.6 | 0.0321 | 0.0078 | 0.0213 | -0.0397 |
|  | b | -17.9 | 0.0264 | 0.0091 | 0.0226 | -0.0248 |
|  | c | -7.4 | 0.0093 | 0.0026 | 0.0288 | -0.0131 |
|  | d | -24.7 | 0.0276 | 0.0071 | 0.0223 | -0.0357 |

**Table S8.** The component values of *α* values (a.u.) for **1-6*[Os]** at various methods.

| Complex | method | *α*_xx_ | *α_yy_* | *α*_zz_ | *α* |
| --- | --- | --- | --- | --- | --- |
| **1*[Os]** | CAM-B3LYP | 173.11 | 193.84 | 165.62 | 177.52 |
|  | BHandHLYP | 171.42 | 193.08 | 162.56 | 175.69 |
|  | M06-2X | 173.85 | 193.99 | 166.79 | 178.21 |
| **2*[Os]** | CAM-B3LYP | 186.26 | 214.80 | 176.33 | 192.46 |
|  | BHandHLYP | 184.69 | 213.70 | 172.88 | 190.43 |
|  | M06-2X | 186.83 | 214.44 | 176.82 | 192.69 |
| **3*[Os]** | CAM-B3LYP | 202.28 | 228.93 | 178.56 | 203.26 |
|  | BHandHLYP | 200.78 | 227.47 | 175.08 | 201.11 |
|  | M06-2X | 202.47 | 228.99 | 179.28 | 203.58 |
| **4*[Os]** | CAM-B3LYP | 206.83 | 282.75 | 181.92 | 223.83 |
|  | BHandHLYP | 205.71 | 286.10 | 178.59 | 223.47 |
|  | M06-2X | 206.89 | 279.72 | 182.90 | 223.17 |
| **5*[Os]** | CAM-B3LYP | 213.04 | 290.85 | 189.13 | 231.01 |
|  | BHandHLYP | 212.25 | 288.44 | 186.29 | 228.99 |
|  | M06-2X | 213.36 | 291.60 | 189.73 | 231.56 |
| **6*[Os]** | CAM-B3LYP | 234.75 | 307.10 | 192.91 | 244.92 |
|  | BHandHLYP | 233.40 | 306.48 | 188.86 | 242.91 |
|  | M06-2X | 234.98 | 307.69 | 192.14 | 244.94 |

**Table S9.The component values of *α* values (a.u.) for 1-6*[Fe] at CAM-B3LYP/def2-TZVPP.**

| Complex | *α*_xx_ | *α_yy_* | *α*_zz_ | *α* |
| --- | --- | --- | --- | --- |
| **1*[Fe]** | 167.07 | 187.84 | 155.91 | 170.27 |
| **2*[Fe]** | 180.99 | 205.42 | 163.78 | 183.40 |
| **3*[Fe]** | 195.33 | 219.98 | 165.45 | 193.59 |
| **4*[Fe]** | 198.35 | 258.12 | 172.29 | 209.59 |
| **5*[Fe]** | 214.85 | 289.87 | 175.46 | 226.73 |
| **6*[Fe]** | 228.40 | 298.01 | 180.41 | 235.61 |

**Table S10. The component values of *α* values (a.u.) for 1-6*[Re] at CAM-B3LYP/def2-TZVPP.**

| Complex | *α*_xx_ | *α_yy_* | *α*_zz_ | *α* |
| --- | --- | --- | --- | --- |
| **1*[Re]** | 178.43 | 196.92 | 174.81 | 183.39 |
| **2*[Re]** | 188.30 | 219.29 | 182.43 | 196.67 |
| **3*[Re]** | 208.03 | 232.69 | 182.75 | 207.82 |
| **4*[Re]** | 208.16 | 274.20 | 186.20 | 222.86 |
| **5*[Re]** | 212.05 | 308.16 | 193.76 | 237.99 |
| **6*[Re]** | 240.41 | 311.51 | 195.46 | 249.12 |

**Table S11.** The component values of *α* values (a.u.) for **1-6*[Ir]** at CAM-B3LYP/def2-TZVPP.

| Complex | *α*_xx_ | *α_yy_* | *α*_zz_ | *α* |
| --- | --- | --- | --- | --- |
| **1*[Ir]** | 168.54 | 186.27 | 167.49 | 174.10 |
| **2*[Ir]** | 182.88 | 201.31 | 172.94 | 185.71 |
| **3*[Ir]** | 202.81 | 213.75 | 175.63 | 197.40 |
| **4*[Ir]** | 193.87 | 261.17 | 178.94 | 211.33 |
| **5*[Ir]** | 219.11 | 298.55 | 184.09 | 233.92 |
| **6*[Ir]** | 229.49 | 293.59 | 189.50 | 237.53 |

**Table S12.** The component values of *γ*_tot_ (a.u.) for **1-6*[Os]** at various methods.

| Complex | method | *γ*_xxxx_ | *γ*_yyyy_ | *γ*_zzzz_ | *γ*_xxyy_ | *γ*_xxzz_ | *γ*_yyzz_ | *γ_tot_* |
| --- | --- | --- | --- | --- | --- | --- | --- | --- |
| **1*[Os]** | CAM-B3LYP | 25800.20 | 12232.50 | 40874.80 | 7844.73 | 5364.95 | 3196.26 | 22343.88 |
|  | BHandHLYP | 25064.70 | 11346.90 | 41223.60 | 7744.57 | 5433.25 | 327.44 | 20929.14 |
|  | M06-2X | 28510.80 | 13756.80 | 40973.30 | 8439.40 | 5597.30 | 3625.30 | 23712.98 |
| **2*[Os]** | CAM-B3LYP | 25864.10 | 12790.60 | 44229.50 | 5264.03 | 4211.83 | 3827.07 | 21898.01 |
|  | BHandHLYP | 25105.20 | 12157.60 | 44399.60 | 5055.32 | 4217.93 | 3797.28 | 21560.69 |
|  | M06-2X | 29054.30 | 15063.10 | 47232.60 | 5785.78 | 4639.47 | 4386.67 | 24194.77 |
| **3*[Os]** | CAM-B3LYP | 24827.20 | 4496.74 | 44943.80 | 12090.20 | 4133.25 | 2849.13 | 22482.58 |
|  | BHandHLYP | 24330.80 | 3523.48 | 45008.00 | 12057.90 | 4190.45 | 2847.28 | 22210.71 |
|  | M06-2X | 26233.10 | 4337.59 | 48279.10 | 12542.70 | 4725.83 | 3465.43 | 24063.54 |
| **4*[Os]** | CAM-B3LYP | 23162.40 | 34144.30 | 44132.30 | 6210.68 | 4492.55 | 3164.81 | 25835.02 |
|  | BHandHLYP | 22376.10 | 37418.00 | 44431.50 | 6994.48 | 4419.23 | 2548.52 | 26430.01 |
|  | M06-2X | 26316.10 | 40060.70 | 50208.80 | 6383.89 | 5842.22 | 4659.85 | 30071.50 |
| **5*[Os]** | CAM-B3LYP | 35760.20 | 133095.00 | 49383.00 | 20874.70 | 6441.96 | 4546.28 | 56392.82 |
|  | BHandHLYP | 36235.40 | 140760.00 | 48457.50 | 22485.60 | 6401.09 | 5195.80 | 58723.58 |
|  | M06-2X | 38235.40 | 160346.00 | 49558.50 | 23369.60 | 6301.09 | 6268.20 | 64003.54 |
| **6*[Os]** | CAM-B3LYP | 31496.00 | 218663.00 | 44304.70 | 9741.73 | 4682.62 | 3929.75 | 66234.38 |
|  | BHandHLYP | 30909.40 | 233382.00 | 44464.30 | 9454.23 | 4656.74 | 3918.59 | 68962.96 |
|  | M06-2X | 33219.80 | 240940.00 | 47592.00 | 9998.43 | 5242.97 | 4477.08 | 72237.75 |

**Table S13.** The component values of *γ*_tot_ (a.u.) for **1-6*[Fe]** at CAM-B3LYP/def2-TZVPP.

| Complex | *γ*_xxxx_ | *γ*_yyyy_ | *γ*_zzzz_ | *γ*_xxyy_ | *γ*_xxzz_ | *γ*_yyzz_ | *γ_tot_* |
| --- | --- | --- | --- | --- | --- | --- | --- |
| **1*[Fe]** | 21125.10 | 11591.80 | 41326.20 | 7011.15 | 5305.67 | 3149.87 | 20995.30 |
| **2*[Fe]** | 21816.20 | 13450.60 | 42852.20 | 4136.42 | 3567.38 | 3798.53 | 20224.73 |
| **3*[Fe]** | 24747.80 | 9031.27 | 42833.20 | 10723.70 | 3577.42 | 3314.14 | 22368.56 |
| **4*[Fe]** | 27527.80 | 25392.60 | 41331.70 | 7807.48 | 4561.12 | 2959.07 | 24981.49 |
| **5*[Fe]** | 30344.60 | 12652.70 | 42965.10 | 11043.40 | 4238.47 | 3064.37 | 24530.98 |
| **6*[Fe]** | 31752.80 | 19252.60 | 42438.70 | 9295.22 | 4007.12 | 2518.35 | 25017.10 |

**Table S14.** The component values of *γ*_tot_ (a.u.) for **1-6*[Re]** at CAM-B3LYP/def2-TZVPP.

| Complex | *γ*_xxxx_ | *γ*_yyyy_ | *γ*_zzzz_ | *γ*_xxyy_ | *γ*_xxzz_ | *γ*_yyzz_ | *γ_tot_* |
| --- | --- | --- | --- | --- | --- | --- | --- |
| **1*[Re]** | 23219.70 | 19437.00 | 54163.30 | 7779.26 | 9323.74 | 3675.06 | 27675.22 |
| **2*[Re]** | 22621.80 | 18961.50 | 54250.00 | 6852.27 | 6493.26 | 4299.47 | 26224.66 |
| **3*[Re]** | 27824.50 | 23947.90 | 55091.70 | 11207.00 | 4556.30 | 3536.42 | 29092.71 |
| **4*[Re]** | 22508.50 | 20565.80 | 47678.80 | 5917.49 | 3322.27 | 2896.33 | 23005.06 |
| **5*[Re]** | 22178.20 | 35761.20 | 55106.40 | 8912.84 | 5366.27 | 3758.84 | 29824.34 |
| **6*[Re]** | 37210.30 | 31518.70 | 51636.20 | 14731.30 | 5501.82 | 4410.73 | 33930.58 |

**Table S15.** The component values of *γ*_tot_ (10^4^ a.u.) for **1-6*[Ir]** at CAM-B3LYP/def2-TZVPP.

| Complex | *γ*_xxxx_ | *γ*_yyyy_ | *γ*_zzzz_ | *γ*_xxyy_ | *γ*_xxzz_ | *γ*_yyzz_ | *γ_tot_* |
| --- | --- | --- | --- | --- | --- | --- | --- |
| **1*[Ir]** | 26566.00 | 26097.40 | 39645.00 | 10725.00 | 4509.24 | 3326.63 | 25886.03 |
| **2*[Ir]** | 30650.80 | 42792.20 | 38230.50 | 10391.80 | 4342.73 | 3776.72 | 29739.20 |
| **3*[Ir]** | 34788.00 | 51240.10 | 39859.80 | 20964.00 | 4882.35 | 3709.37 | 36999.87 |
| **4*[Ir]** | 32124.70 | 39620.30 | 37716.00 | 19591.80 | 4464.93 | 3641.84 | 32971.63 |
| **5*[Ir]** | 32411.40 | 13544.60 | 40815.20 | 11609.20 | 4186.59 | 3575.54 | 25102.77 |
| **6*[Ir]** | 46427.00 | 130107.00 | 39124.30 | 23978.80 | 4638.33 | 4083.46 | 56211.90 |

**Table S16.** The transition energies ($\Delta E_{ge}$, eV), oscillator strengths and $f_{os}^{2}/\Delta E_{ge}^{5}$ values (10^-5^) of complexes calculated at the CAM-B3LYP/def2-TZVPP level.

| Complex | state | $\Delta E_{ge}$ | $f_{\text{os}}$ | $f_{os}^{2}/\Delta E_{ge}^{5}$ |
| --- | --- | --- | --- | --- |
| **1*[Os]** | S4 | 3.24 | 0.073 | 1.33 |
| **2*[Os]** | S4 | 3.13 | 0.069 | 1.38 |
| **3*[Os]** | S1 | 2.02 | 0.023 | 1.57 |
| **4*[Os]** | S2 | 1.98 | 0.024 | 2.74 |
| **5*[Os]** | S4 | 1.86 | 0.059 | 15.61 |
| **6*[Os]** | S1 | 1.99 | 0.070 | 15.83 |

**
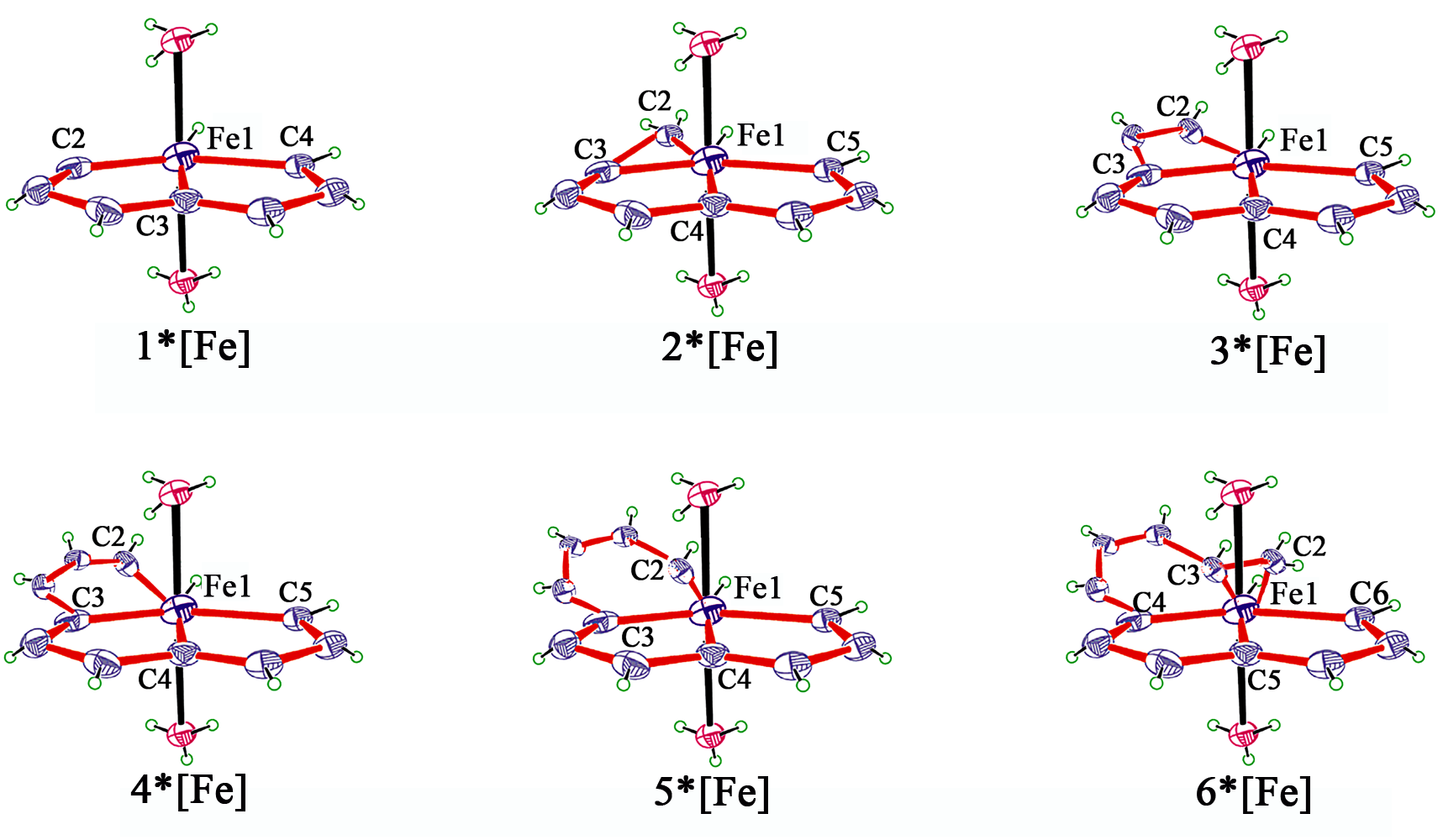
**

**Figure S1.** The Structural models of **1-6*[Fe]**.


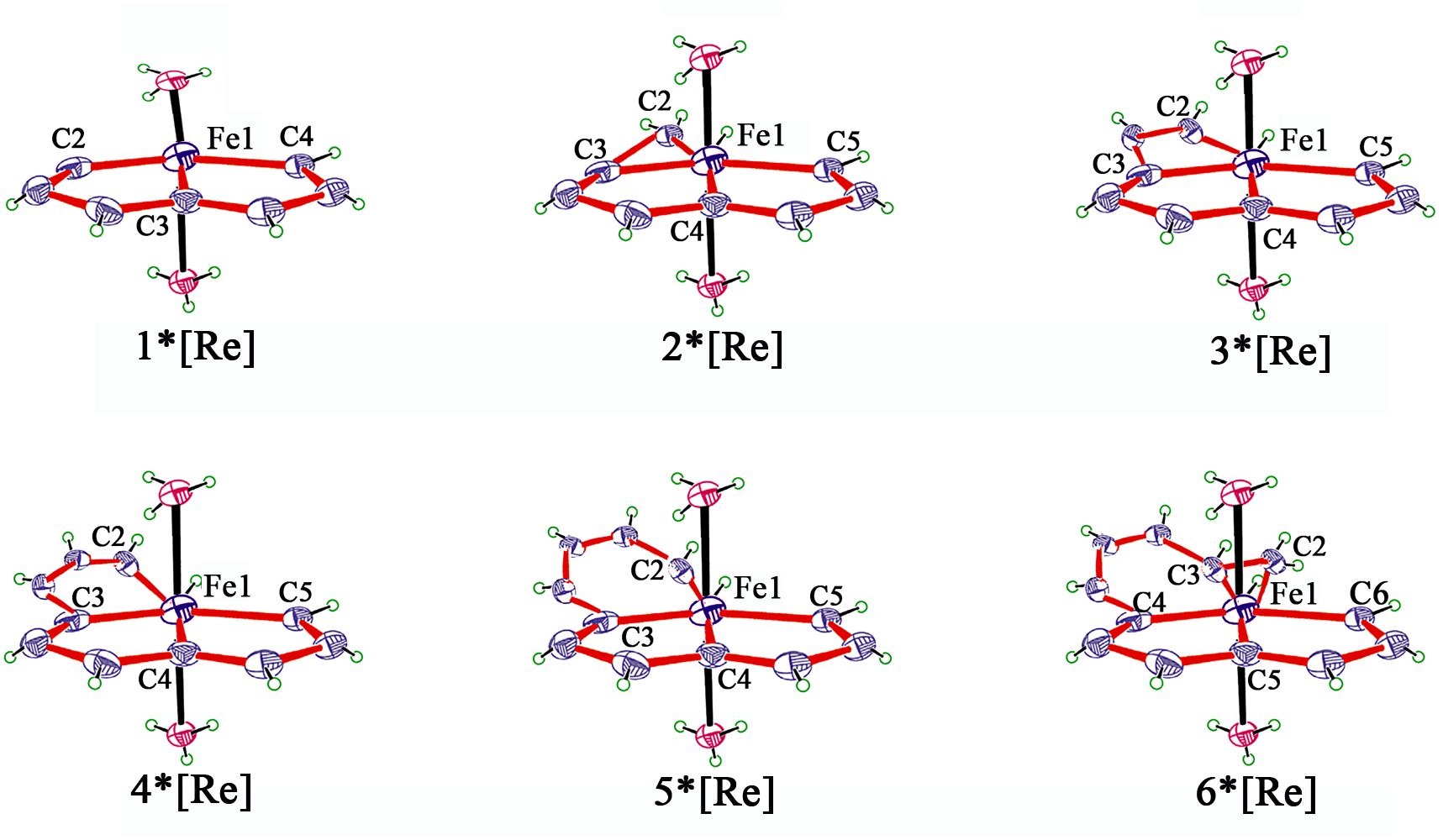


**Figure S2.** The Structural models of **1-6*[Re]**.


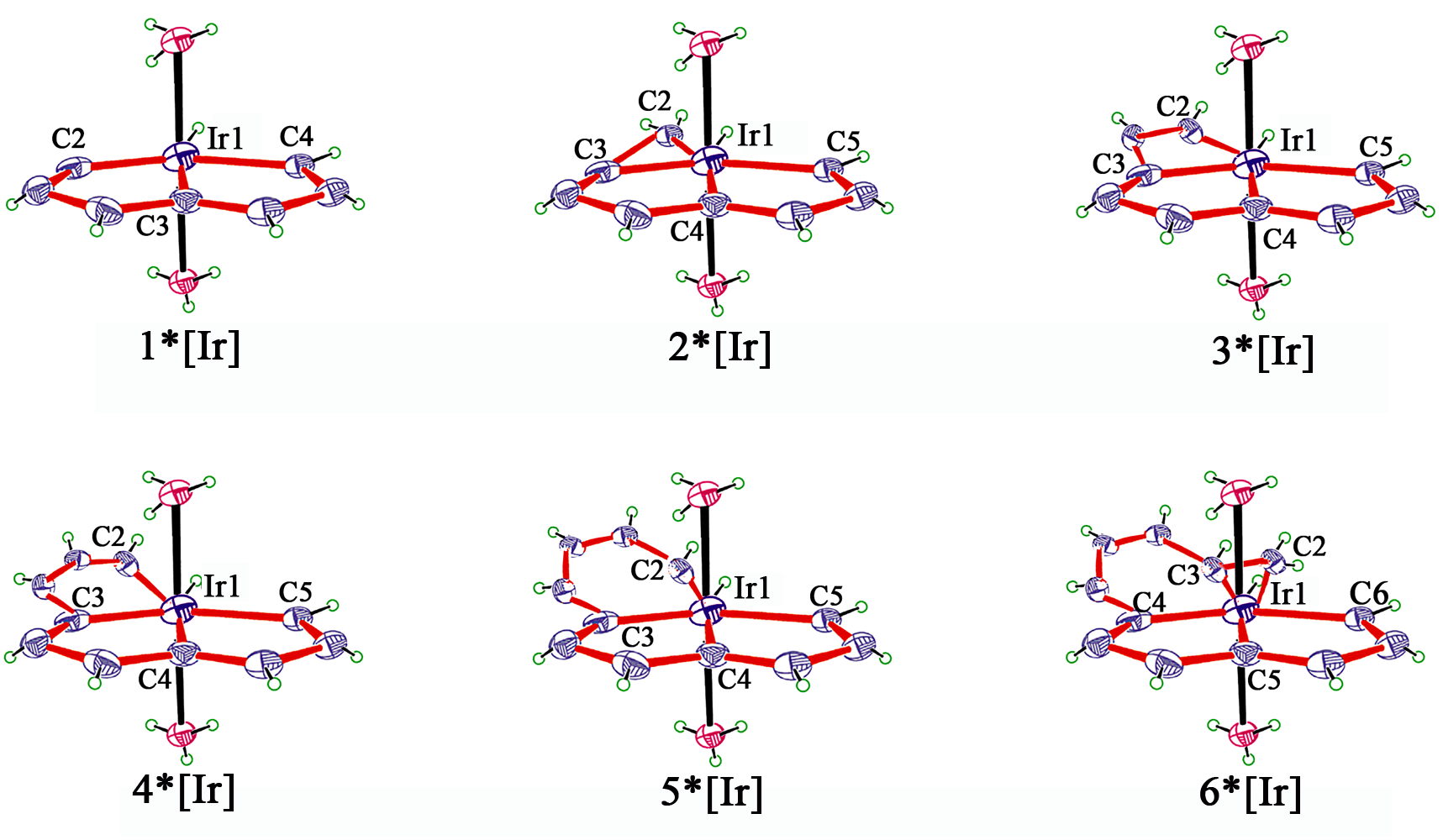


**Figure S3.** The Structural models of **1-6*[Ir]**.


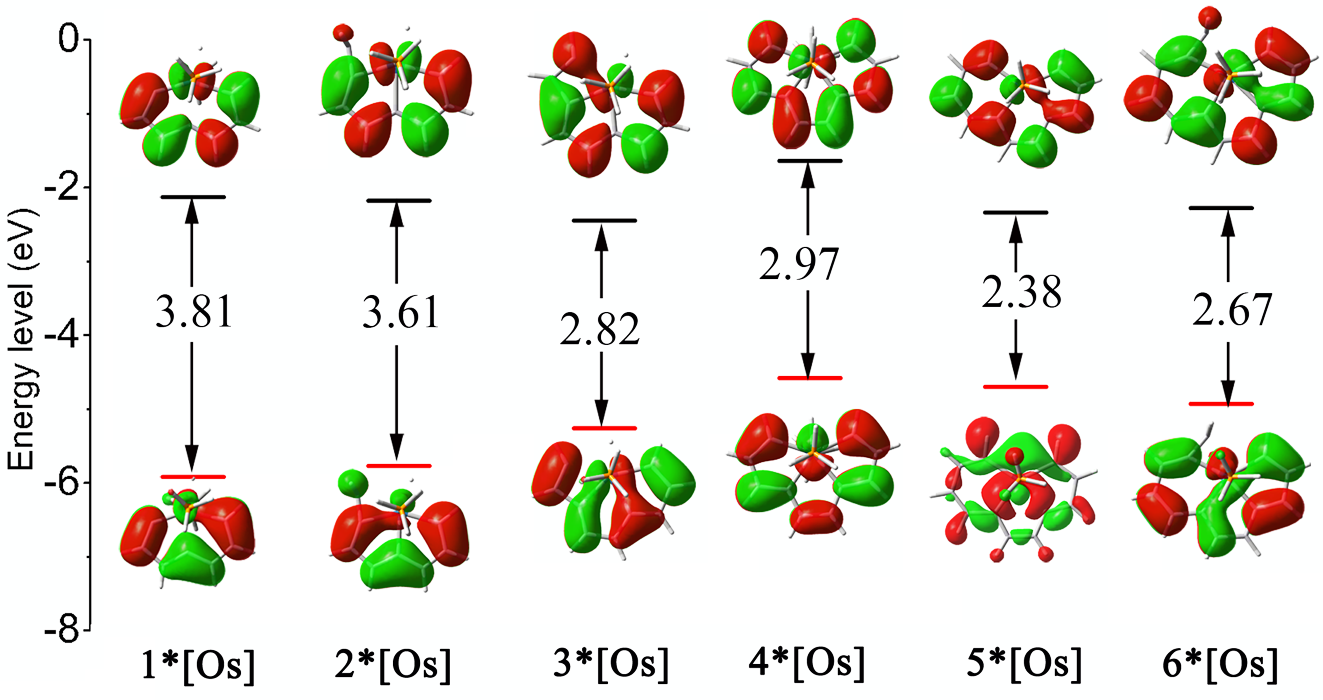


**Figure S4.** The energies and diagrams of frontier molecular orbitals for **1-6*[Os]**.


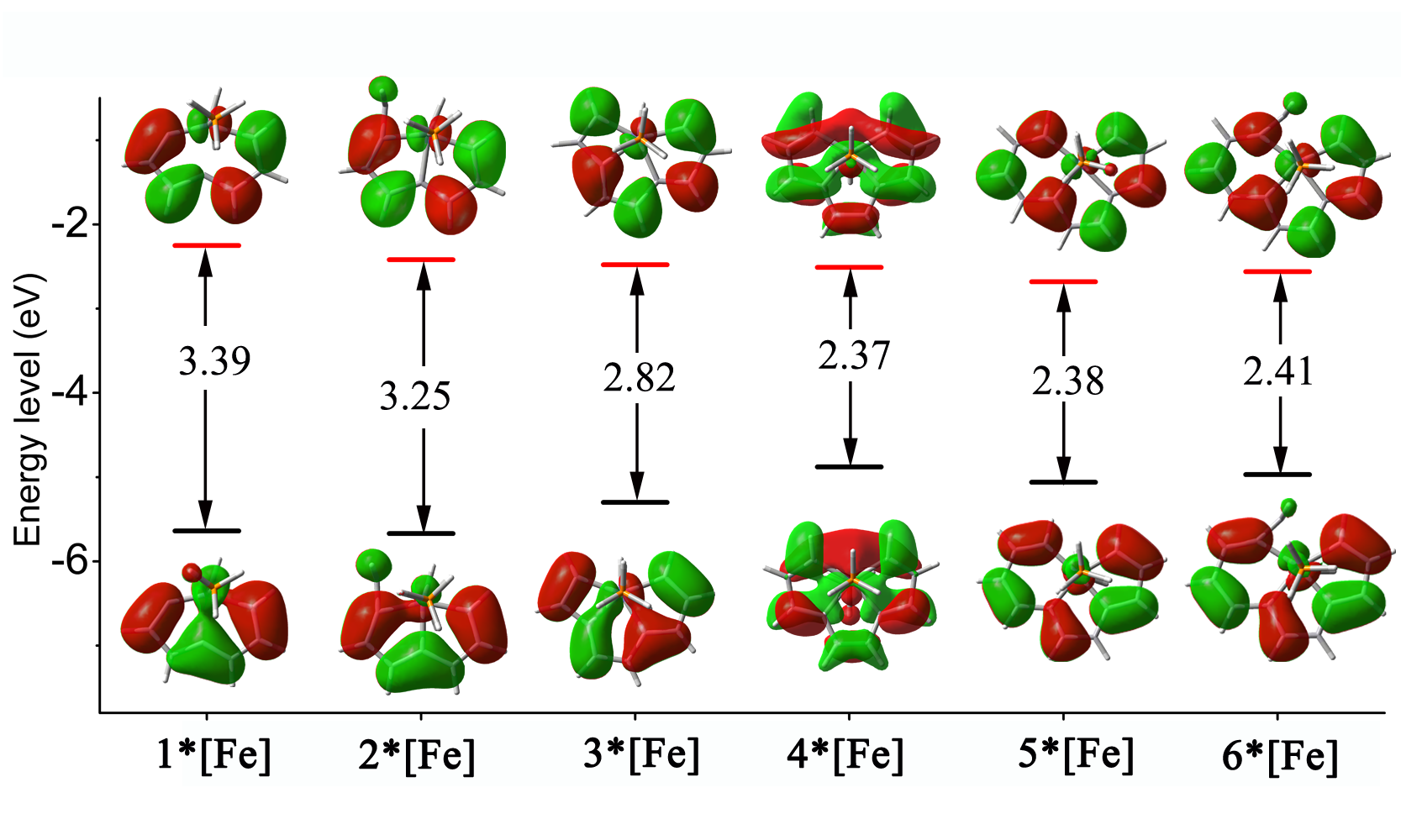


**Figure S5.** The frontier molecular orbital diagrams of complexes **1-6*[Fe]**.


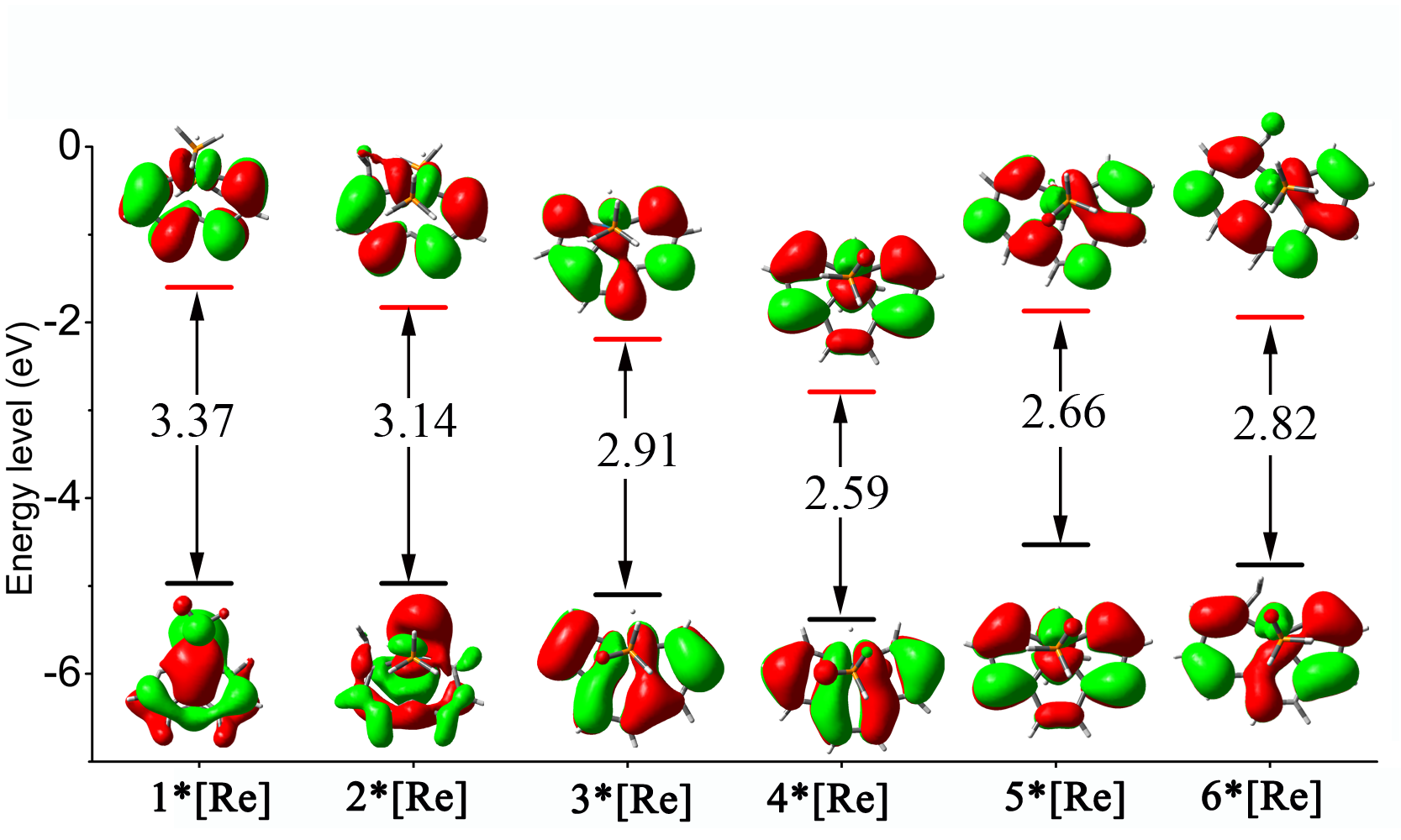


**Figure S6.** The frontier molecular orbital diagrams of complexes **1-6*[Re]**.


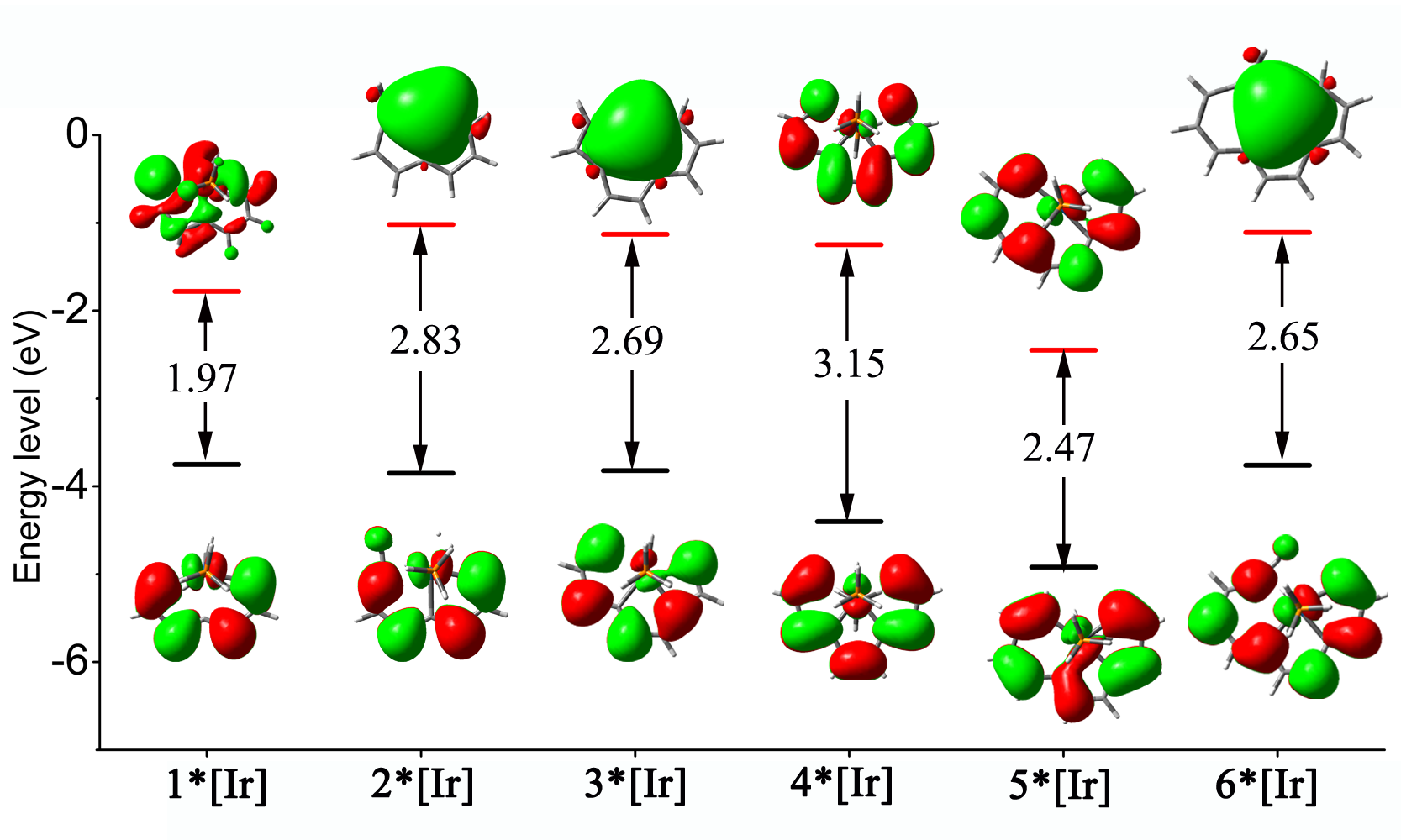


**Figure S7.** The frontier molecular orbital diagrams of complexes **1-6*[Ir]**.


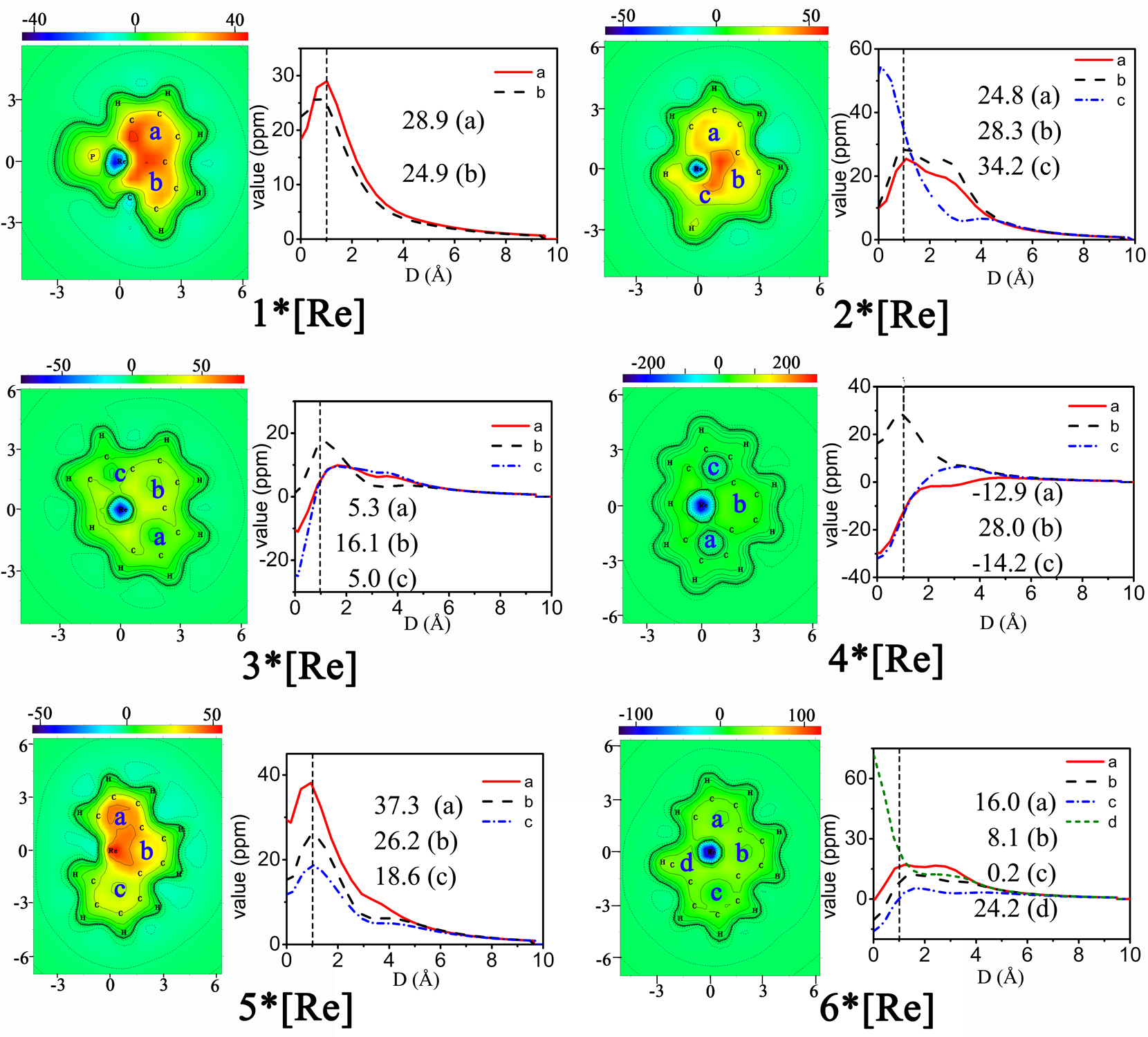


**Figure S8.** The visualize cut-plane map of ICSS_ZZ_ and –NICS_ZZ_ values of **1-6*[Re]** at ring centers by scanning its value in a line.


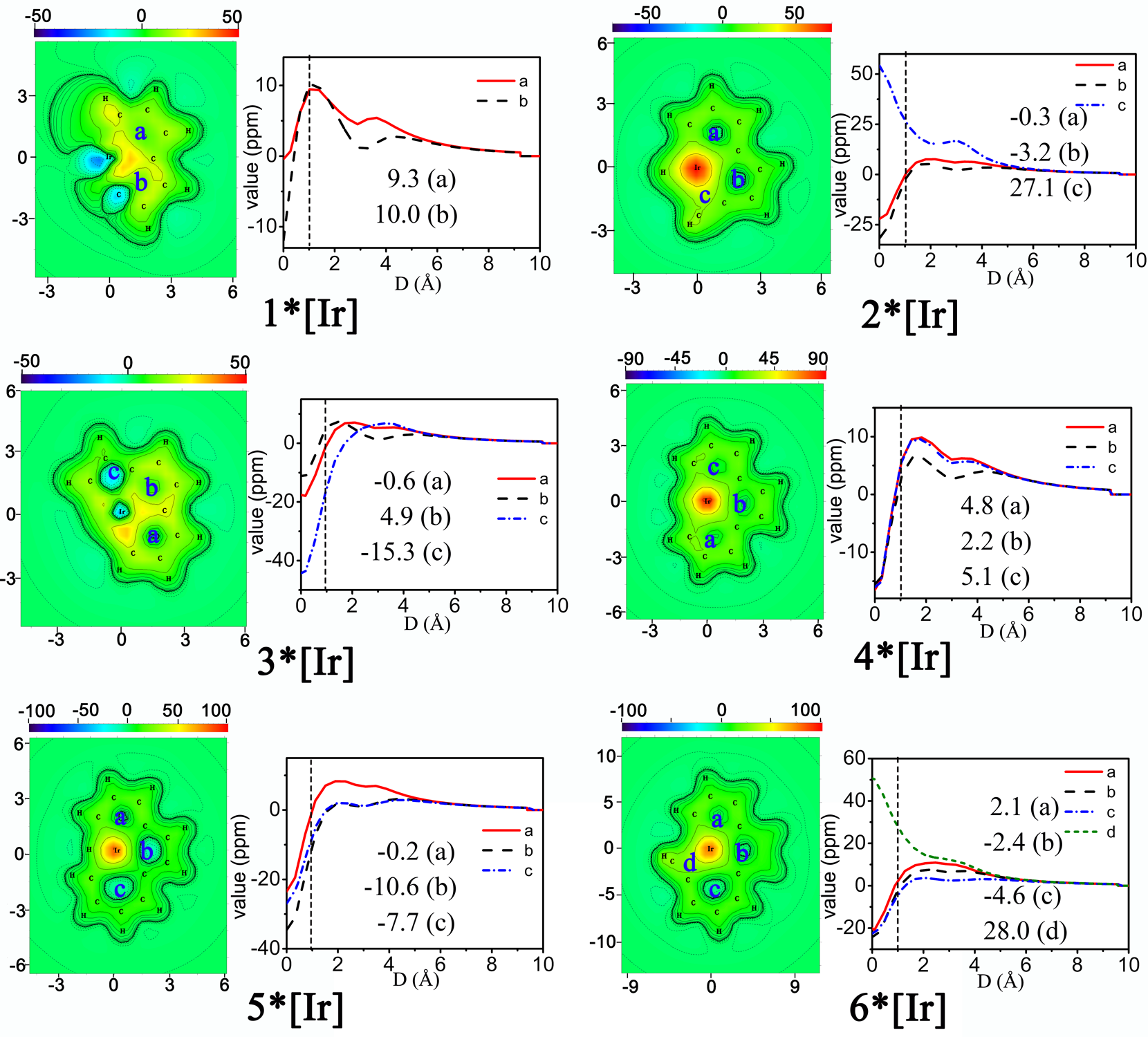


**Figure S9.** The visualize cut-plane map of ICSS_ZZ_ and –NICS_ZZ_ values of **1-6*[Ir]** at ring centers by scanning its value in a line.


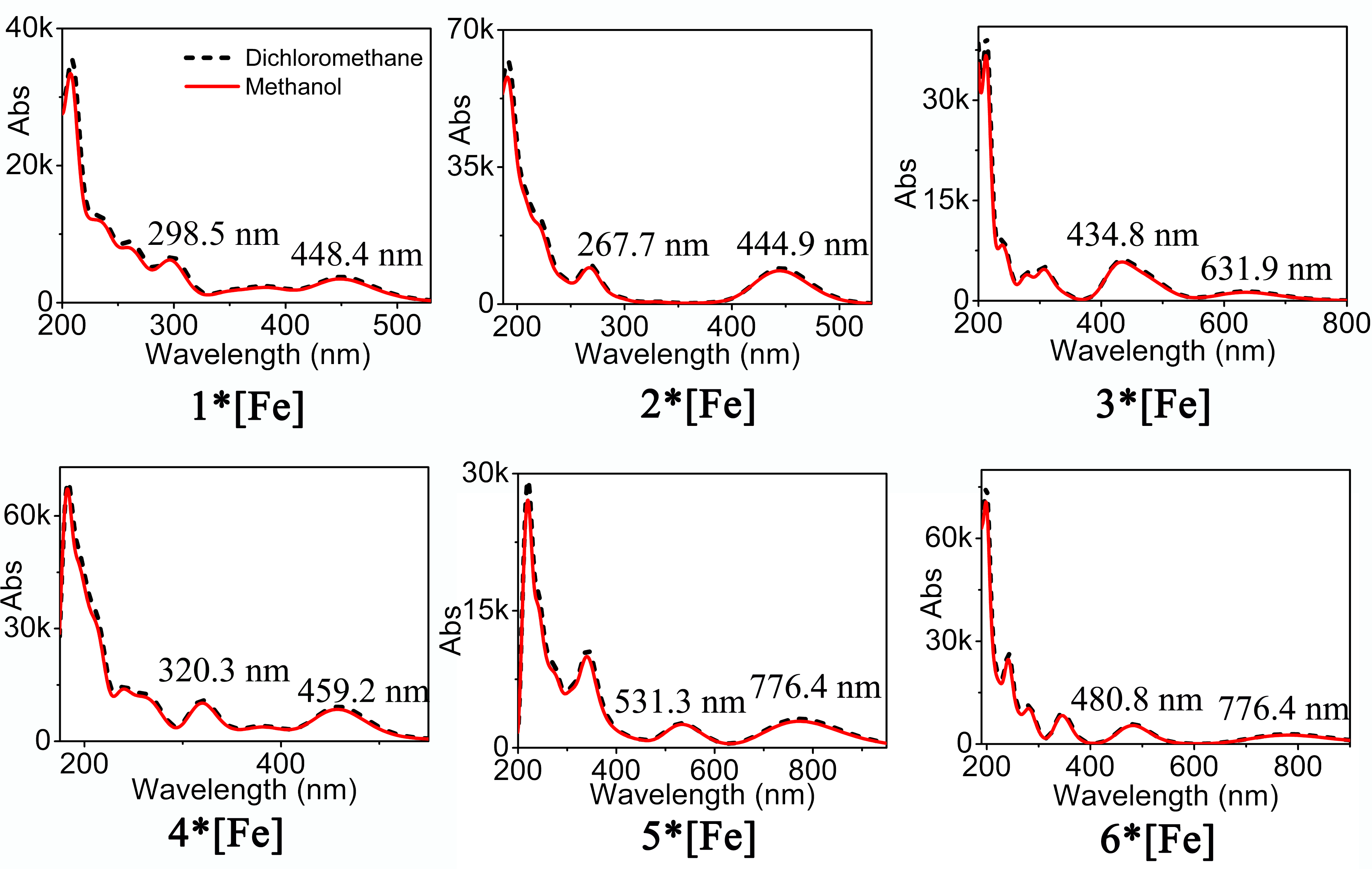


**Figure S10.** Absorption spectra (Abs: A is absorbance coefficient, L/(g·cm). b is the layer thickness, cm. c is solution concentration, g/L.) of the complexes **1-6*[Fe]** in the solvents of dichloromethane and methanol.


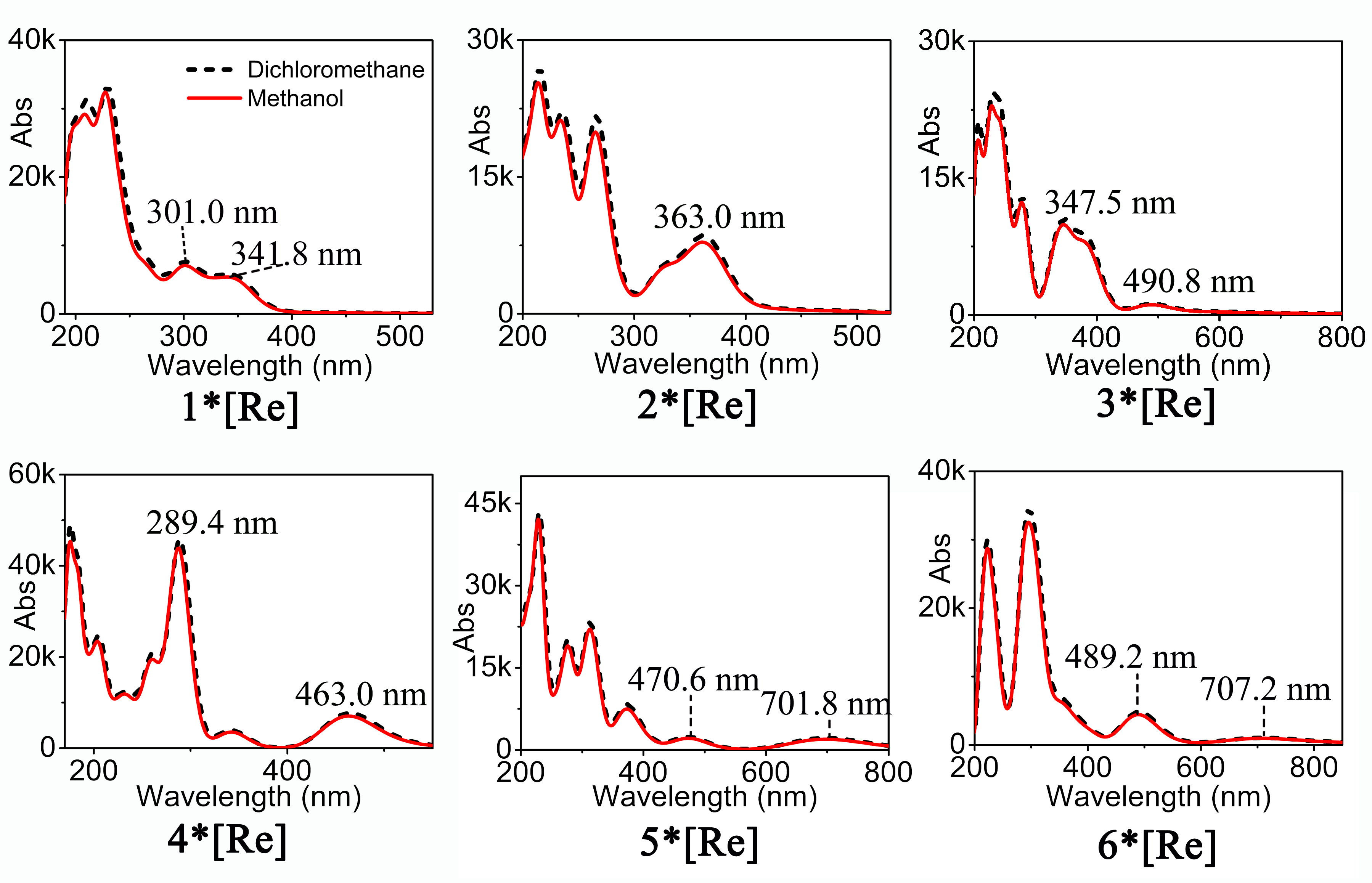


**Figure S11.** Absorption spectra (Abs: A is absorbance coefficient, L/(g·cm). b is the layer thickness, cm. c is solution concentration, g/L.) of the complexes **1-6*[Re]** in the solvents of dichloromethane and methanol.


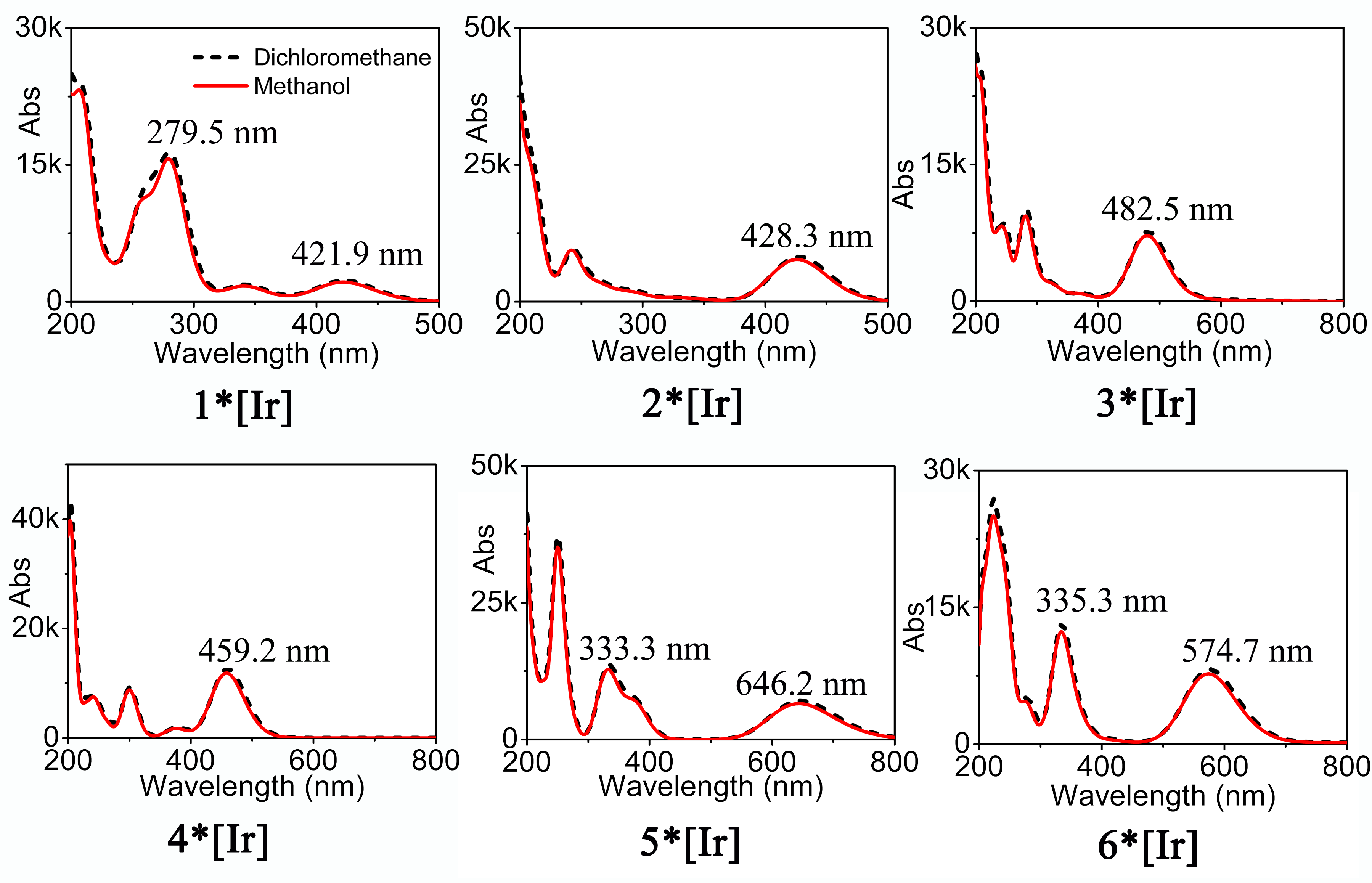


**Figure S12.** Absorption spectra (Abs: A is absorbance coefficient, L/(g·cm). b is the layer thickness, cm. c is solution concentration, g/L.) of the complexes **1-6*[Ir]** in the solvents of dichloromethane and methanol.
